# Supplementary material for: Air-stable 18-electron adducts of Schrock catalysts with tuned stability constants for spontaneous release of the active species
Source: Commun Chem. 2021 May 19;4:71. doi: 10.1038/s42004-021-00503-4 (PMC9814263; doi:10.1038/s42004-021-00503-4)
Supplement: Supplementary file 2 — Description of Additional Supplementary Files [file 42004_2021_503_MOESM2_ESM.pdf]

Description of Additional Supplementary Files

File Name: Supplementary Data 1

Description: Crystallographic Information File (cif) for compound 8

File Name: Supplementary Data 2

Description: Crystallographic Information File (cif) for compound 9
