# Supplementary material for: Air-stable 18-electron adducts of Schrock catalysts with tuned stability constants for spontaneous release of the active species
Source: Commun Chem. 2021 May 19;4:71. doi: 10.1038/s42004-021-00503-4 (PMC9814263; doi:10.1038/s42004-021-00503-4)
Supplement: Supplementary file 3 — Supplementary Information [file 42004_2021_503_MOESM3_ESM.pdf]

Air-Stable 18-Electron Adducts of Schrock Catalysts with Tuned Stability Constants for Spontaneous Release of the Active Species

Henrik Gulyás,[1]* Shigetaka Hayano,[2] Ádám Madarász,[3] Imre Pápai,[3]* Márk Szabó,[1] Ágota Bucsai,[1] Eddy Martin,[4, 5] Jordi Benet-Buchholz[4]

[1]*XiMo Hungary Ltd, Berlini Park, Budapest, Hungary*

[2]*Zeon Corporation, Tokyo, Japan*

[3] *Research Center for Natural Sciences, Institute of Organic Chemistry, Budapest, Hungary*

[4] *Institute of Chemical Research of Catalonia (ICIQ), Barcelona Institute of Science and Technology (BIST), Tarragona, Spain*

[5]*Bruker France SAS, Wissembourg, France*

Supplementary Information

Supplementary Methods

1. Synthesis and characterization of tungsten and molybdenum complexes

1.1. General

All reactions were carried in N_2 or Ar filled gloveboxes, using oven-dried glassware (120 – 150 °C, 5 – 24 hours).

All reagents derived from commercial sources (abcr, Alfa Aesar, Fluca, Fluorchem, Sigma-Aldrich) were used as received unless otherwise specified.

Activated molecular sieves (3 Å, beads, 4-8 mesh) were used to fine-dry (fully dehydrate) pre-dried bulk solvents, deuterated solvents, and certain reagent solutions. Bulk solvents, after drying them by conventional methods, were kept in gloveboxes over activated molecular sieves. Deuterated solvents were kept in the glovebox over molecular sieves. The molecular sieves were activated in high vacuum ($\leq 10^{-2}$ mbar), at 200 °C, for 16 hours.

Bulk solvents first were dried via conventional methods before transferring them into the glovebox and fine-drying them with activated molecular sieves. Benzene, toluene, Et_2O, THF were distilled from potassium under N_2 atmosphere, and then they were stored over activated molecular sieves in gloveboxes. DCM was distilled from CaH_2 under N_2 atmosphere, and then it was stored over activated molecular sieves in gloveboxes. C_6D_6, d8-toluene, and CD_2Cl_2 were dried by adding activated molecular sieves to them at least 12 hours before using them. They were stored over the added molecular sieves in a glovebox.

Commercial 1,10'-phenanthroline and 2,2'-bipyridine were dehydrated in DCM solution over activated molecular sieves. The molecular sieves were filtered off, and DCM was evaporated in high vacuum. The recovery was typically between 85 – 98%.

$Mo(NAr^{diiPr})(CHCMe_2Ph)(OCMe(CF_3)_2)_2$ (**1**), $Mo(NAr^{diiPr})(CHCMe_2Ph)(OCMe(CF_3)_2)_2$(1,10-phenantroline) (**2**), $Mo(NAr^{diiPr})(CHCMe_2Ph)(OCMe(CF_3)_2)_2$(2,2'-bipyridine) (**3**), $W(NAr^{diiPr})($ $CHCMe_2Ph)(OSiPh_3)_2$ (**4**) and $W(NAr^{diiPr})(CHCMe_2Ph)(2,5\text{-}Me_2NC_4H_2)(OCPh(CF_3)_2)$ (**5**) (ArdiiPr = 2,6-diisopropyl-phenyl) were prepared according to literature methods.[1,2]

NMR characterization of all new compounds was carried out on a narrow bore Ultrashield Bruker NMR spectrometer with an Avance console operating at 300 MHz, using 5 mm ^1H-^{13}C DUI and ^{19}F-^1H SEL probes. Pulse sequences from TopSpin 1.3 software were used without modification. The sample was

temperature-controlled by heated airflow with an accuracy of 0.1 K. Chemical shifts (δ) are given in ppm relative to $SiMe_4$ (^{1}H and ^{13}C) and $CFCl_3$ (^{19}F). Coupling constants (J) are given in Hz. Typically, solvent signals were used as references to determine chemical shifts (in C_6D_6, C_6D_6 for ^{13}C: $\delta_C \equiv 128.06$ ppm, and residual C_6D_5H for ^{1}H: $\delta_H \equiv 7.16$ ppm).

HRMS ESI-TOF measurements were carried out on LC 1200 Series coupled to MS 6210 Time of Flight (Agilent Technologies). The following injection conditions were used for all measurements, sample volume: 2 microL, mobile phase: acetonitrile, flow rate: 0.6 mL/min. The ESI conditions applied were the following, capillary voltage: 3500 V, gas temperature: 350 °C, drying gas flow: 12 L/min, nebulizer gas pressure: 60 psi. The TOF conditions used were the following, mode: positive ion, fragmentor voltage: 150 V, skimmer voltage: 65 V, octapole voltage: 250 V, acquisition: from 50 to 1200 m/z.

Single-crystal X-ray diffraction studies were performed using Bruker Nonius and Bruker Kappa Apex II Duo diffractometers equipped with an APEX II 4K CCD area detector and an Oxford Cryostream low-temperature device at 100 K.

1.2. Synthesis of W(NAr^{diiPr})(CHCMe$_2$Ph)(OSiPh$_3$)$_2$(1,10-phenanthroline) (Ar^{diiPr} = 2,6-diisopropyl-phenyl; 6)

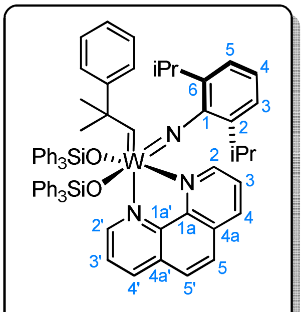

6

W(CHCMe$_2$Ph)(NAr)(OSiPh$_3$)$_2$ (Ar = 2,6-diisopropyl-phenyl) (208 mg, 0.2 mmol) and 1,10-phenanthroline (36 mg, 0.2 mmol) were transferred into a vial. Toluene (2 mL) was added. A deep red homogeneous solution was formed immediately. The solution was stirred at RT for 2 hours. Pentane was slowly added (ca. 2 mL in total). The vial was transferred into the freezer (-40 Celsius). The product crystallized from the solution by the following morning. It was filtered off, washed with pentane, and dried in high vacuum. Orange solid. Yield: 174 mg (71%). The mother liqueur was transferred into the

freezer again. By the following morning, a considerable amount of crystals were deposited on the walls of the vial again. They were preserved for possible X-ray analysis.

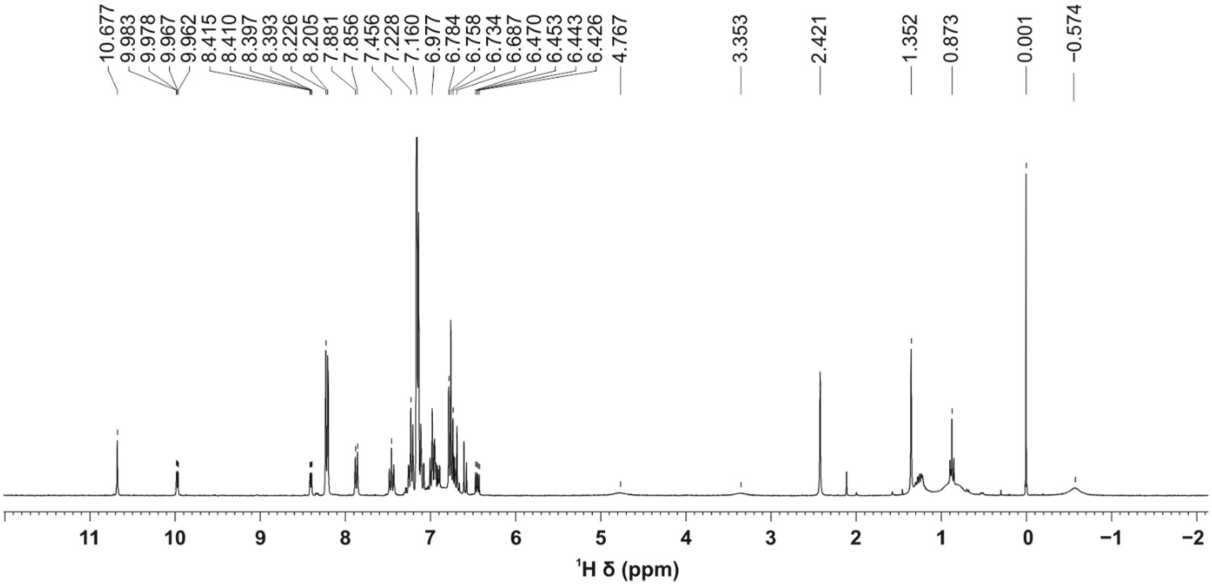

Supplementary Figure 1. ^{1}H NMR spectrum of **6** in C_6D_6 (*c* = 0.05 M) at 298 K

^{1}H NMR (300 MHz, C_6D_6, 25°C): δ -0.56 (br, 3H, NAr 2-iPr $CH_3$1); 0.77 (br, 3H, NAr 2-iPr $CH_3$2); 0.89 (br, 3H, NAr 6-iPr $CH_3$1); 1.26 (br, 3H, NAr 6-iPr $CH_3$2); 1.35 (s, 3H, neoph $CH_3$1); 2.42 (s, 3H, neoph $CH_3$2); 3.35 (br, 1H, NAr 2-iPr CH); 4.78 (br, 1H, NAr 6-iPr CH); 6.45 (dd, $^{3}J_{H\text{-}H}$ = 8.3, 5.4 Hz, 1H, phen 3); 6.58 (d, $^{3}J_{H\text{-}H}$ = 7.6, 2H, NAr 3, 5); 6.68 (overlapped, 1H, Si-Ph^{1} para); 6.70 (t, $^{3}J_{H\text{-}H}$ = 7.6 Hz, 1H, NAr 4); 6.77 (overlapped, 2H, phen 5, 5'); 6.91 (dd, $^{3}J_{H\text{-}H}$ = 8.0, 4.9 Hz, 1H, phen 3'); 6.99 (dd, $^{3}J_{H\text{-}H}$ = 8.3, $^{4}J_{H\text{-}H}$ = 1.5 Hz, 1H, phen 4); 7.12 (dd, $^{3}J_{H\text{-}H}$ = 8.0, $^{4}J_{H\text{-}H}$ = 1.7 Hz, 1H, phen 4'); 7.15 (overlapped, 2H, Si-Ph^{1} meta); 7.22 (t, $^{3}J_{H\text{-}H}$ = 7.6 Hz, 1H, neoph para); 7.46 (t, $^{3}J_{H\text{-}H}$ = 7.6 Hz, 2H, neoph meta); 7.86 (d, $^{3}J_{H\text{-}H}$ = 7.6 Hz, 2H, neoph ortho); 8.21 (dd, $^{3}J_{H\text{-}H}$ = 6.8, $^{4}J_{H\text{-}H}$ = 1.6 Hz, 2H, Si-Ph^{1} ortho); 8.39 (dd, $^{3}J_{H\text{-}H}$ = 5.4, $^{4}J_{H\text{-}H}$ = 1.5 Hz, 1H, phen 2); 9.97 (dd, $^{3}J_{H\text{-}H}$ = 4.9, $^{4}J_{H\text{-}H}$ = 1.7 Hz 1H, phen 2'); 10.68 (t, $^{2}J_{W\text{-}H}$ = 6.6 Hz, 1H, W=CH) ppm.

^{13}C NMR (75.5 MHz, C_6D_6, 25°C): δ 20.0-28.7 (br, 6C, NAr 2-iPr $CH_3$1, $CH_3$2, CH; 6-iPr $CH_3$1, $CH_3$2, CH); 29.0 (neoph $CH_3$2); 34.5 (neoph $CH_3$1); 54.8 (neoph C); 123.4 (phen 3'); 124.0 (NAr 4); 124.1 (phen 3); 124.1 (2C, Si-Ph^{1} para); 126.4 (NAr 3, 5); 126.6 (phen 5, 5'); 126.7 (2C, neoph ortho); 128.2 (2C, neoph meta); 128.6 (neoph para); 135.2 (2C, Si-Ph^{1} meta); 135.7 (phen 4a); 136.4 (phen 4'); 136.7 (2C, Si-Ph^{1} ortho); 137.4 (phen 4); 138.6 (phen 4a'); 142.9 (phen 1a'); 144.5 (phen 1a); 146.4 (NAr 2); 148.6 (phen 2'); 149.9 (NAr 1); 150.5 (NAr 6); 155.9 (neoph ipso); 158.7 (phen 2); 269.3 (W=CH) ppm.

HRMS (ESI^+): m/z calculated for $[M + K]^+$: 1260.3918, found: 1260.3891; calculated for $[M + Na]^+$: 1244.4178, found: 1244.4175; calculated for $[M – OSiPh_3]^+$: 946.3389, found: 946.3393.

1.3. Synthesis of W(NAr^{diiPr})($CHCMe_2Ph$)(2,5-$Me_2NC_4H_2$)($OCPh(CF_3)_2$)(1,10-phenanthroline) (Ar^{diiPr} = 2,6-diisopropyl-phenyl; 7)

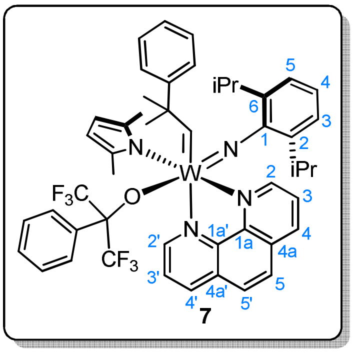

The bispyrrolide precursor W($CHCMe_2Ph$)(NAr^{diiPr})(Me_2Pyr)$_2$ (Ar^{diiPr} = 2,6-diisopropylphenyl, Me_2Pyr = 2,5-dimethylpyrrolide) (680 mg, 1 mmol) was dissolved in benzene (10 mL). $Ph(CF_3)_2COH$ (170 microL, 1 mmol) was added, and the reaction mixture was stirred for an hour at room temperature. An aliquot of the reaction mixture was analyzed by ^1H and ^{19}F NMR, which confirmed the formation of the MAP complex. 1,10-Phenantroline (180 mg, 1 mmol) was added. The reaction mixture was stirred for an hour at room temperature. An aliquot was analyzed by ^1H and ^{19}F NMR. Both methods confirmed the formation of one single stereoisomer of the target compound. The compound was spectroscopically pure. The solution of the complex was concentrated to ca. 3-4 mL. Pentane was added to room temperature. Precipitation of the target compound started after adding ca. 15 mL pentane. Some more pentane (ca. 10 mL) was added to complete precipitation. The mixture was stirred for an hour. The solids were isolated by filtration, washed with pentane, and dried first in N_2 stream, and then in vacuum. Ocher solid. Yield: 936 mg (93%). The product was prepared from the isolated MAP complex, too, with similar yields.

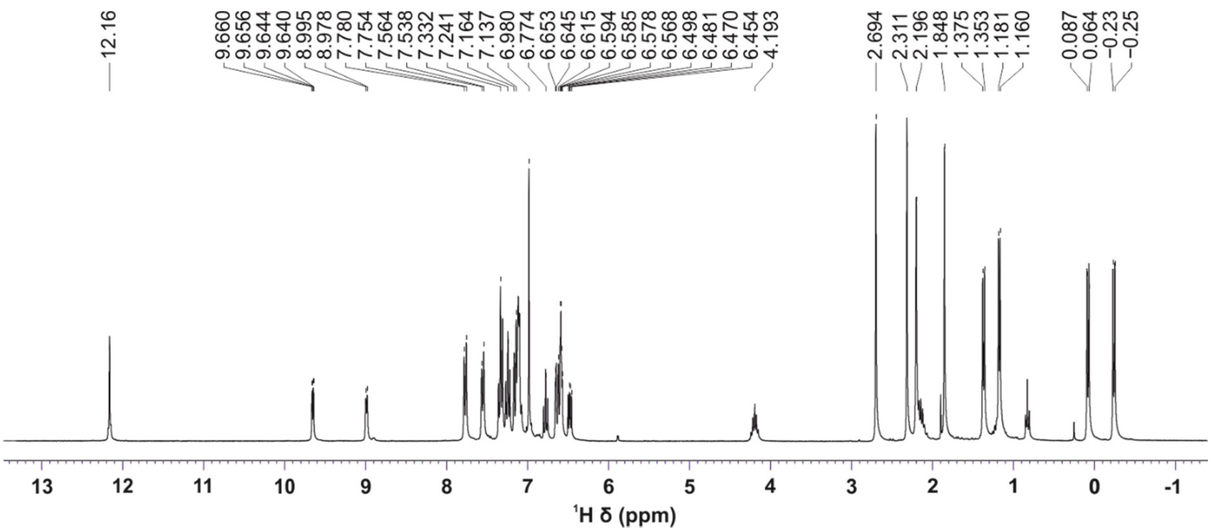

Supplementary Figure 2. ^{1}H NMR spectrum of **7** in C_6D_6 (*c* = 0.05 M) at 298 K

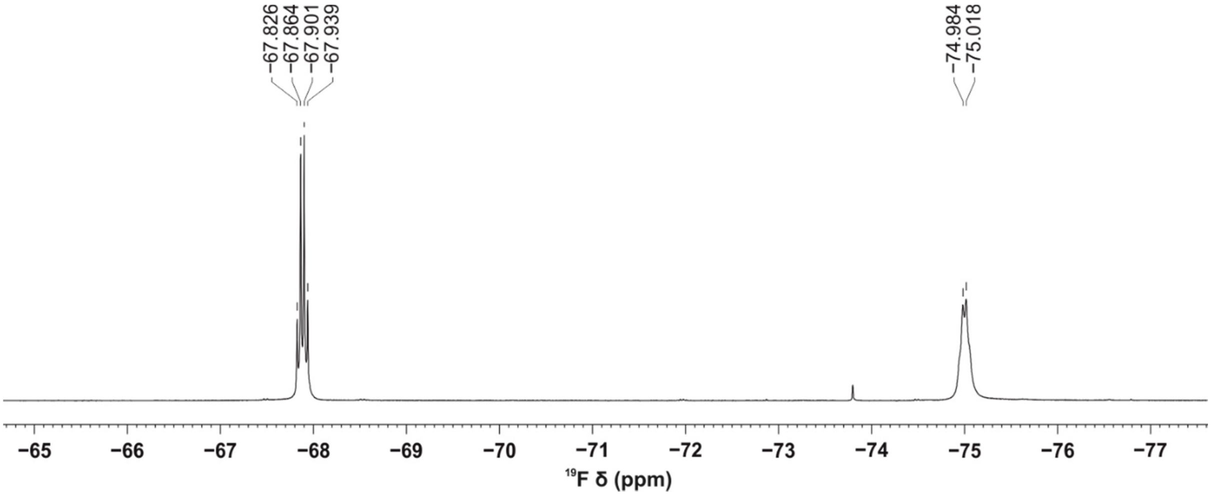

Supplementary Figure 3. ^{19}F NMR spectrum of **7** in C_6D_6 (*c* = 0.05 M) at 298 K

^{1}H NMR (300 MHz, C_6D_6, 25°C): δ -0.24 (d, $^{3}J_{H\text{-}H}$ = 6.9 Hz, 3H, NAr 2-iPr $CH_3$1); 0.08 (d, 3H, $^{3}J_{H\text{-}H}$ = 6.9 Hz, NAr 2-iPr $CH_3$2); 1.18 (d, $^{3}J_{H\text{-}H}$ = 6.5 Hz, 3H, NAr 6-iPr $CH_3$1); 1.37 (d, $^{3}J_{H\text{-}H}$ = 6.5 Hz, 3H, NAr 6-iPr $CH_3$2); 1.85 (s, 3H, neoph $CH_3$1); 2.14 (septet, $^{3}J_{H\text{-}H}$ = 6.9 Hz, 1H, NAr 2-iPr CH); 2.20 (s, 3H, neoph $CH_3$2); 2.32 (s, 3H, Me_2Pyr 2-CH_3); 2.70 (s, 3H, Me_2Pyr 5-CH_3); 4.20 (septet, $^{3}J_{H\text{-}H}$ = 6.6 Hz, 1H, NAr 6-iPr CH); 6.48 (dd, $^{3}J_{H\text{-}H}$ = 8.1, 5.0 Hz, 1H, phen 3'); 6.58 (d, $^{3}J_{H\text{-}H}$ = 2.5, 1H, Me_2Pyr 4-CH); 6.60 (dd, $^{3}J_{H\text{-}H}$ = 7.8, 5.1 Hz, 1H, phen 3); 6.61 (dd, $^{3}J_{H\text{-}H}$ = 7.7, $^{4}J_{H\text{-}H}$ = 1.2 Hz, 1H, NAr 3); 6.65 (d, $^{3}J_{H\text{-}H}$ = 2.5 Hz, 1H, Me_2Pyr 3-CH); 6.78 (t, $^{3}J_{H\text{-}H}$ = 7.7 Hz, 1H, NAr 4); 6.99 (AB d, 2H, phen 5, 5'); 7.10 (t, $^{3}J_{H\text{-}H}$ = 7.8 Hz, 1H, $OC(CF_3)_2$Ph para); 7.11 (dd, $^{3}J_{H\text{-}H}$ = 7.7, $^{4}J_{H\text{-}H}$ = 1.2 Hz, 1H, NAr 5); 7.14 (t, $^{3}J_{H\text{-}H}$ = 7.7 Hz, 1H, neoph para); 7.16 (d, $^{3}J_{H\text{-}H}$ = 8.1 Hz, 1H, phen 4'); 7.25 (t, $^{3}J_{H\text{-}H}$ = 7.8 Hz, 2H, $OC(CF_3)_2$Ph meta); 7.32 (d, $^{3}J_{H\text{-}H}$ = 7.8 Hz, 1H, phen 4); 7.34 (t, $^{3}J_{H\text{-}H}$ = 7.7 Hz, 2H, neoph meta); 7.56 (d, $^{3}J_{H\text{-}H}$ = 7.8 Hz, 2H, $OC(CF_3)_2$Ph ortho); 7.78 (d, $^{3}J_{H\text{-}H}$ = 7.7

Hz, 2H, neoph ortho); 8.99 (d, $^{3}J_{H\text{-}H}$ = 5.1 Hz, 1H, phen 2); 9.66 (dd, $^{3}J_{H\text{-}H}$ = 8.1, $^{4}J_{H\text{-}H}$ = 1.3 Hz 1H, phen 2'); 12.16 (t, $^{2}J_{W\text{-}H}$ = 9.5 Hz, 1H, W=CH) ppm.

^{19}F NMR (282.4 MHz, C_6D_6, 25°C): δ -75.0 (q, $^{4}J_{F\text{-}F}$ = 10.6 Hz, 3F, $CF_3$1); -67.88 (q, $^{4}J_{F\text{-}F}$ = 10.6 Hz, 3F, $CF_3$2).

^{13}C NMR (75.5 MHz, C_6D_6, 25°C): 21.9 (Me_2Pyr 5-CH_3); 22.1 (Me_2Pyr 2-CH_3); 22.8 (NAr 2-iPr $CH_3$1); 23.9 (NAr 2-iPr $CH_3$2); 25.0 (NAr 6-iPr $CH_3$1); 27.5 (NAr 6-iPr $CH_3$2); 28.0 (NAr 2-iPr CH); 28.1 (NAr 6-iPr CH); 30.9 (neoph $CH_3$2); 36.5 (neoph $CH_3$1); 55.0 (neoph C); 83.9 ($O\underline{C}(CF_3)_2Ph$); 109.0 (Me_2Pyr 3-CH); 112.3 (Me_2Pyr 4-CH); 122.2 (NAr 3); 124.5 (phen 3'); 124.7 (phen 3); 125.3 (NAr 5); 126.1 (NAr 4); 126.4 (phen 5); 126.6 (neoph para); 127.3 (2C, neoph ortho); 127.6 (phen 5'); 128.5 ($OC(CF_3)_2Ph$ meta); 128.8 (2C, neoph meta); 129.1 ($OC(CF_3)_2Ph$ ortho); 129.1 ($OC(CF_3)_2Ph$ para); 134.0 (Me_2Pyr 2); 136.3 ($OC(CF_3)_2Ph$ ipso); 137.8 (phen 4'); 138.7 (Me_2Pyr 5); 139.2 (phen 4); 145.7 (phen 4a'); 146.5 (NAr 2); 147.2 (phen 4a); 150.7 (NAr 6); 152.3 (NAr 1); 153.8 (neoph ipso); 153.9 (phen 2'); 153.9 (phen 1a'); 154.6 (phen 1a); 161.4 (phen 2); 285.8 (W=CH) ppm.

HRMS (ESI^{+}): m/z calculated for $[M - OC(CF_3)_2Ph]^{+}$: 765.3154, found: 765.3147; calculated for $[M - (2,5\text{-}Me_2NC_4H_2)]^{+}$: 914.2741, found: 914.2738.

1.4. Synthesis of W(NAr^{diiPr})($CHCMe_2Ph$)($OSiPh_3$)$_2$(2,2'-bipyridine) (Ar^{diiPr} = 2,6-diisopropyl-phenyl; 8)

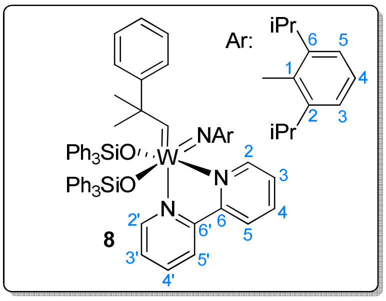

W($CHCMe_2Ph$)(NAr)($OSiPh_3$)$_2$ (Ar = 2,6-diisopropyl-phenyl) (417 mg, 0.4 mmol) and 2,2'-bipyridine (62.5 mg, 0.4 mmol) were transferred into a vial. Toluene (3 mL) was added. A deep red homogeneous solution was formed immediately. The solution was stirred at RT overnight. Pentane (2.5 mL) was added, and the vial was transferred into the freezer. No crystallization could be initiated, the compound precipitated as an oil. The vial was removed from the fridge, and left in the box at room temperature. The crystallization of the product started spontaneously in a few hours. The crystals

were isolated by filtration, washed with small amounts of cold pentane, and dried in vacuum induced N_2 flow. Deep orange crystals. Yield: 436 mg (91%).

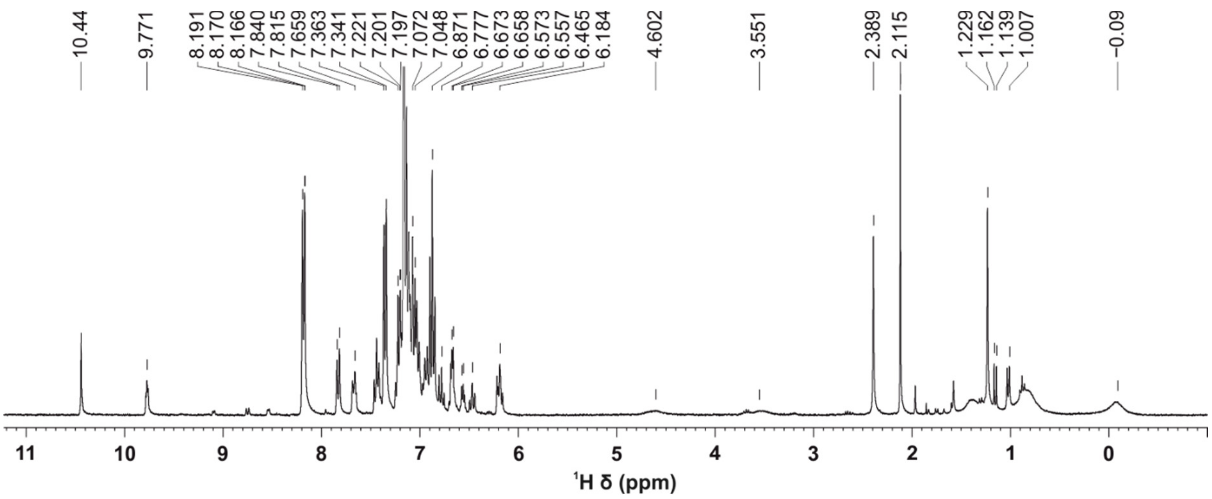

Supplementary Figure 4: ^{1}H NMR spectrum of **8** in C_6D_6 (*c* = 0.03 M) at 298 K

^{1}H NMR (300 MHz, C_6D_6, 25°C): δ -0.06 (br, 3H, NAr 2-iPr $CH_3$1); 0.83 (br, 3H, NAr 2-iPr $CH_3$2); 0.89 (br, 3H, NAr 6-iPr $CH_3$1); 1.23 (s, 3H, neoph $CH_3$1); 1.38 (br, 3H, NAr 6-iPr $CH_3$2); 2.39 (s, 3H, neoph $CH_3$2); 3.54 (br, 1H, NAr 2-iPr CH); 4.61 (br, 1H, NAr 6-iPr CH); 6.19 (dd, $^3J_{H\text{-}H}$ = 7.9, 5.6 Hz, 1H, bipy 3); 6.20 (overlapped, 1H, bipy 5); 6.47 (td, $^3J_{H\text{-}H}$ = 7.9, $^4J_{H\text{-}H}$ = 1.4 Hz, 1H, bipy 4); 6.56 (overlapped, 1H, bipy 5'); 6.93 (d, $^3J_{H\text{-}H}$ = 7.6, 2H, NAr 3, 5); 6.67 (m, 1H, bipy 4'); 6.68 (m, 1H, bipy 3'); 6.77 (t, $^3J_{H\text{-}H}$ = 7.6 Hz, 1H, NAr 4); 6.87 (t, $^3J_{H\text{-}H}$ = 7.8 Hz, 6H, Si-Ph^1 meta); 7.14 (t, $^3J_{H\text{-}H}$ = 7.5 Hz, 6H, Si-Ph^2 meta); 7.20 (t, $^3J_{H\text{-}H}$ = 8.0 Hz, 1H, neoph para); 7.35 (d, $^3J_{H\text{-}H}$ = 7.8 Hz, 6H, Si-Ph^1 orthoH); 7.44 (t, $^3J_{H\text{-}H}$ = 8.0 Hz, 2H, neoph meta); 7.83 (d, $^3J_{H\text{-}H}$ = 8.0 Hz, 2H, neoph ortho); 8.18 (d, $^3J_{H\text{-}H}$ = 7.5 Hz, 6H, Si-Ph^2 ortho); 8.18 (overlapped, 1H, bipy 2); 9.77 (br dd, 1H, bipy 2'); 10.44 (t, $^2J_{W\text{-}H}$ = 6.4 Hz, 1H, W=CH) ppm.

^{13}C NMR (75.5 MHz, C_6D_6, 25°C): δ 29.1 (neoph $CH_3$2); 34.7 (neoph $CH_3$1); 51.9 (neoph C); 120.9 (bipy 5'); 121.4 (bipy 5); 122.6 (2C, NAr 3, 5); 124.1 (NAr 4); 124.5 (bipy 3'); 124.9 (bipy 3); 125.1 (neoph para); 126.8 (2C, neoph ortho); 126.8 (6C, Si-Ph^1 meta); 127.3 (6C, Si-Ph^2 meta); 127.9 (2C, neoph meta); 135.9 (6C, Si-Ph^1 ortho); 136.7 (6C, Si-Ph^2 ortho); 137.2 (bipy 4'); 137.7 (bipy 4); 146.3 (NAr 6); 148.6 (bipy 2'); 150.1 (NAr 1); 150.3 (NAr 2); 151.4 (bipy 6'); 152.8 (bipy 6); 155.6 (neoph ipso); 159.3 (bipy 2); 269.4 (W=CH) ppm.

HRMS (ESI^+): m/z calculated for $[M – OSiPh_3]^+$: 922.3389, found: 922.3394.

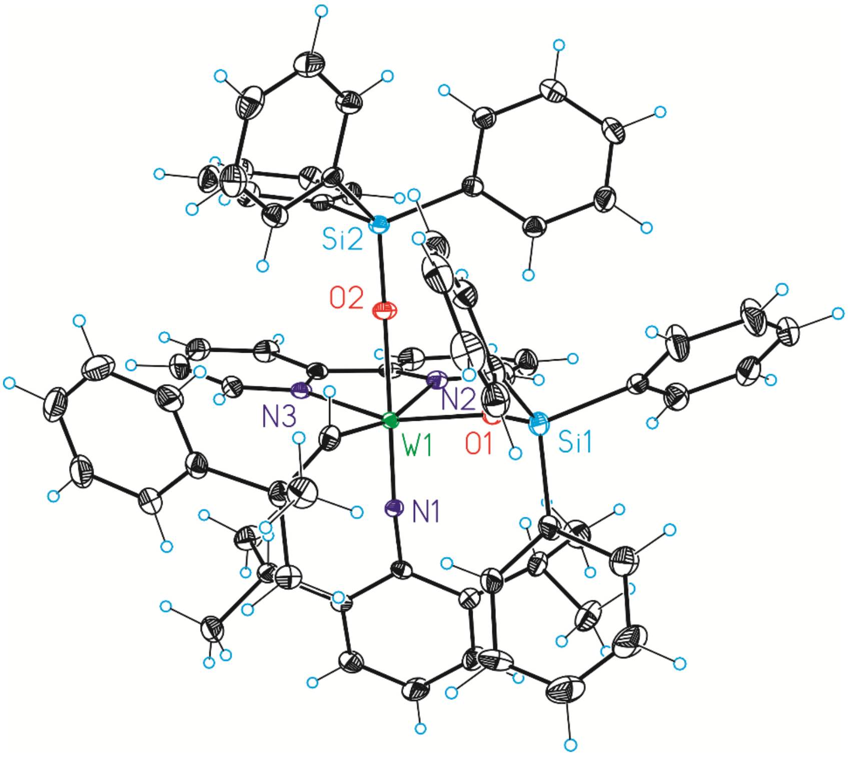

Supplementary Figure 5. Solid-state structure of $W(NAr^{diiPr})(CHCMe_2Ph)(OSiPh_3)_2$(2,2'-bipyridine) (**8**) determined by single-crystal X-ray diffraction; the asymmetric unit contains one molecule of the metal complex and one molecule of toluene (omitted for clarity); one of the phenyl rings of one of the $SiPh_3$ moiety is disordered in two orientations (ratio: 60:40); the structure is of good quality with an R1 value of 3.07 %; for further structural details see the corresponding section

1.5. Synthesis of W(NAr^{diiPr})(CHCMe$_2$Ph)(2,5-Me$_2$NC$_4$H$_2$)(OCPh(CF$_3$)$_2$)(2,2’-bipyridine) (Ar^{diiPr} = 2,6-diisopropyl-phenyl; 9)

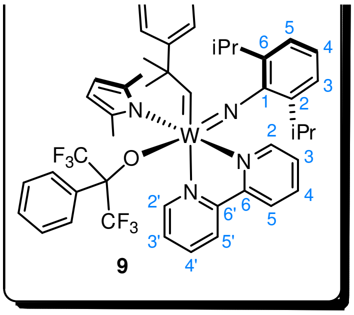

The bispyrrolide precursor W(CHCMe$_2$Ph)(NAr^{diiPr})(Me$_2$Pyr)$_2$ (Ar^{diiPr} = 2,6-diisopropylphenyl, Me$_2$Pyr = 2,5-dimethylpyrrolide) (170 mg, 0.25 mmol) was dissolved in toluene (3 mL). Ph(CF$_3$)$_2$COH (61 mg, 42 microL, 0.25 mmol) was added. The residues from the vial of the alcohol were rinsed into the reaction vial with toluene (2x1 mL). The reaction mixture was stirred for 3 hours at room temperature. An aliquot of the reaction mixture was analyzed by ^{1}H and ^{19}F NMR. Both methods indicated complete conversion into the desired MAP complex. 2,2’-Bipyridine (39 mg, 0.25 mmol) was added as a solid. The reaction mixture turned dark orange immediately. The residues from the vial of the bipyridine were rinsed into the reaction vial with toluene (2x1 mL). The reaction mixture was stirred for 20 minutes at room temperature, and then the solvent was slowly evaporated *in vacuo*. The resulting dark orange solids were washed with pentane. The solids were isolated by filtration and washed with small amounts of cold pentane. The product was dried first in vacuum induced N$_2$ stream, and then in a high vacuum. Orange solid. Yield: 205 mg (83%).

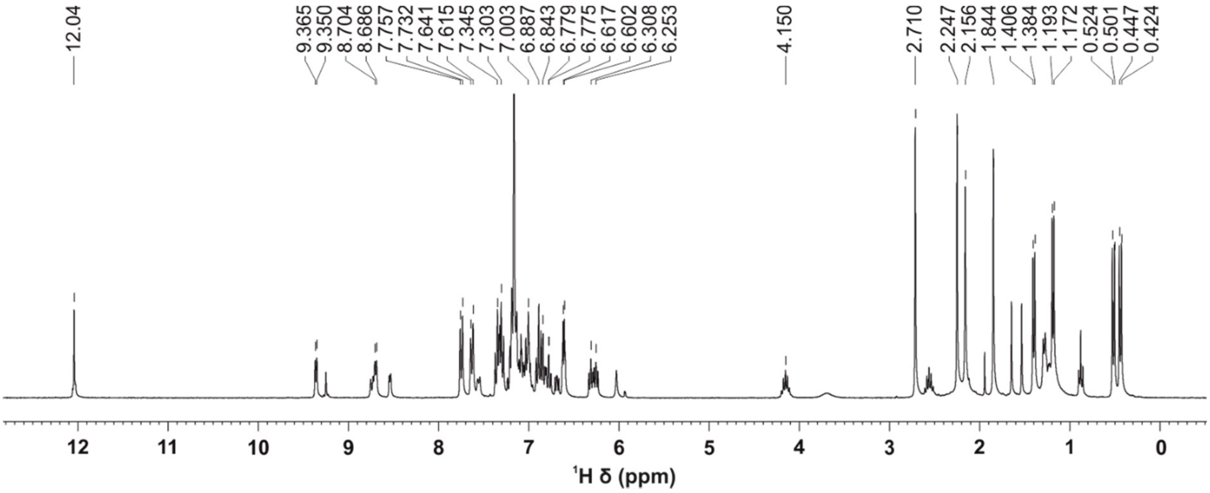

Supplementary Figure 6. ^{1}H NMR spectrum of **9** in C_6D_6 (*c* = 0.05 M) at 298 K

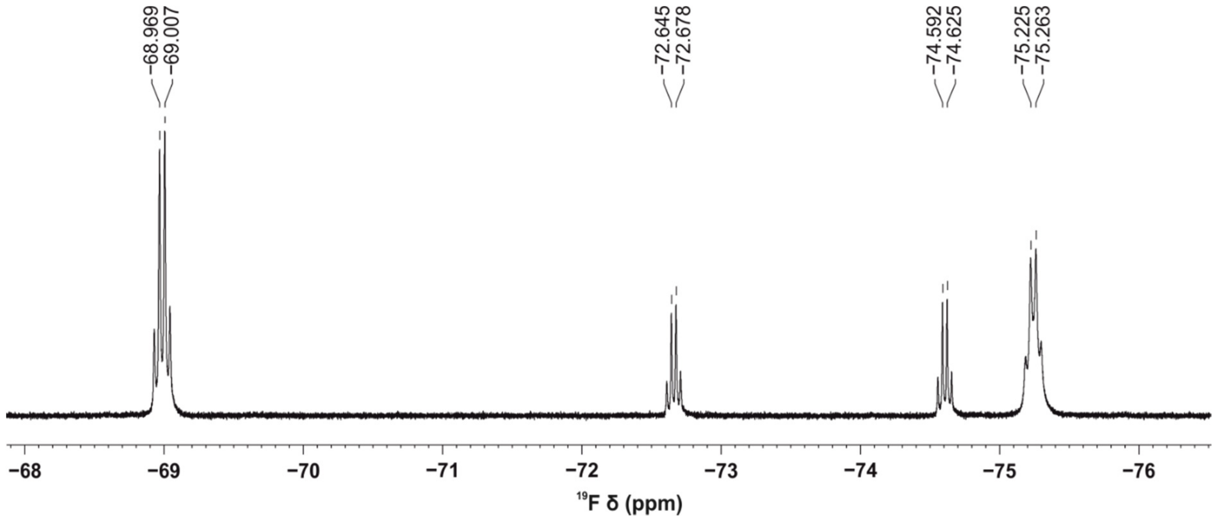

Supplementary Figure 7. ^{19}F NMR spectrum of **9** in C_6D_6 (c = 0.05 M) at 298 K

^{1}H NMR (300 MHz, C_6D_6, 25°C): δ 0.44 (d, $^3J_{H\text{-}H}$ = 7.0 Hz, 3H, NAr 2-iPr $CH_3$1); 0.53 (d, 3H, $^3J_{H\text{-}H}$ = 7.0 Hz, NAr 2-iPr $CH_3$2); 1.19 (d, $^3J_{H\text{-}H}$ = 6.6 Hz, 3H, NAr 6-iPr $CH_3$1); 1.40 (d, $^3J_{H\text{-}H}$ = 6.6 Hz, 3H, NAr 6-iPr $CH_3$2); 1.85 (s, 3H, neoph $CH_3$1); 2.16 (s, 3H, neoph $CH_3$2); 2.25 (s, 3H, 2-Me_2Pyr CH_3); 2.56 (septet, $^3J_{H\text{-}H}$ = 7.0 Hz, 1H, NAr 2-iPr CH); 2.71 (s, 3H, 5-Me_2Pyr CH_3); 4.15 (septet, $^3J_{H\text{-}H}$ = 6.6 Hz, 1H, NAr 6-iPr CH); 6.60 (AB d, 1H, 4-Me_2Pyr CH); 6.61 (AB d, 1H, 3-Me_2Pyr CH); 6.25 (ddd, $^3J_{H\text{-}H}$ = 6.6, 5.5, $^4J_{H\text{-}H}$ = 1.0 Hz, bipy 3'); 6.31 (ddd, $^3J_{H\text{-}H}$ = 7.2, 5.7 $^4J_{H\text{-}H}$ = 1.3 Hz, bipy 3); 6.78 (ddd, $^3J_{H\text{-}H}$ = 6.6, 5.5, $^4J_{H\text{-}H}$ = 1.2 Hz, bipy 4'); 6.89 (t, $^3J_{H\text{-}H}$ = 7.7 Hz, 1H, NAr 4); 6.89 (t, $^3J_{H\text{-}H}$ = 7.2, bipy 4); 7.00 (overlapped, 1H, bipy 5'); 7.13 (d, $^3J_{H\text{-}H}$ = 7.2, bipy 5); 7.16 (m, 1H, neoph para); 7.16 (m, 1H, $OC(CF_3)_2$Ph para); 7.18 (d, $^3J_{H\text{-}H}$ = 7.7 Hz, 1H, NAr 5); 7.19 (d, $^3J_{H\text{-}H}$ = 7.7 Hz, 1H, NAr 3); 7.31 (m, 2H, $OC(CF_3)_2$Ph meta); 7.35 (t, $^3J_{H\text{-}H}$ = 7.5 Hz, 1H, neoph meta); 7.63 (m, 2H, $OC(CF_3)_2$Ph ortho); 7.75 (d, $^3J_{H\text{-}H}$ = 7.5 Hz, 1H, neoph ortho); 8.70 (d, $^3J_{H\text{-}H}$ = 5.7 Hz, 1H, bipy 2); 9.36 (dd, $^3J_{H\text{-}H}$ = 5.5, $^4J_{H\text{-}H}$ = 1.2 Hz, 1H, bipy 2'); 12.04 (t, $^2J_{W\text{-}H}$ = 9.9 Hz, 1H, W=CH) ppm.

^{19}F NMR (282.4 MHz, C_6D_6, 25°C): δ -75.24 (q, $^4J_{F\text{-}F}$ = 10.6 Hz, 3F, $CF_3$1); -68.99 (q, $^4J_{F\text{-}F}$ = 10.6 Hz, 3F, $CF_3$2) ppm.

^{13}C NMR (75.5 MHz, C_6D_6, 25°C): δ 21.0 (2-Me_2Pyr CH_3); 21.2 (5-Me_2Pyr CH_3); 23.3 (2C, NAr 2-iPr1 CH_3); 24.2 (NAr 6-iPr $CH_3$1); 26.8 (NAr 6-iPr $CH_3$2); 27.3 (NAr 6-iPrCH); 27.5 (NAr 3-iPrCH); 29.9 (neoph $CH_3$2); 35.7 (neoph $CH_3$1); 54.2 (neoph C); 108.2 (4-Me_2Pyr CH); 111.5 (3-Me_2Pyr CH); 120.5 (bipy 5'); 120.8 (bipy 5); 121.6 (NAr 3); 124.7 (NAr 5); 124.7 (bipy 3); 124.9 (bipy 3'); 125.6 (neoph para); 126.5 (neoph ortho); 127.7 ($OC(CF_3)_2$Ph meta); 127.8 (neoph meta); 128.2 ($OC(CF_3)_2$Ph para); 128.4 ($OC(CF_3)_2$Ph ortho); 138.0 (bipy 4'); 139.0 (bipy 4); 145.9 (NAr 2); 150.2 (NAr 6); 151.4 (NAr 1); 152.9 (bipy 2'); 152.9 (bipy 6'); 153.8 (neoph ipso); 155.4 (bipy 6); 161.4 (bipy 2); 285.0 (W=CH) ppm.

HRMS (ESI$^+$): m/z calculated for $[M - OC(CF_3)_2Ph]^+$: 741.3154, found: 741.3156; calculated for $[M - (2,5\text{-}Me_2NC_4H_2)]^+$: 890.2741, found: 890.2734.

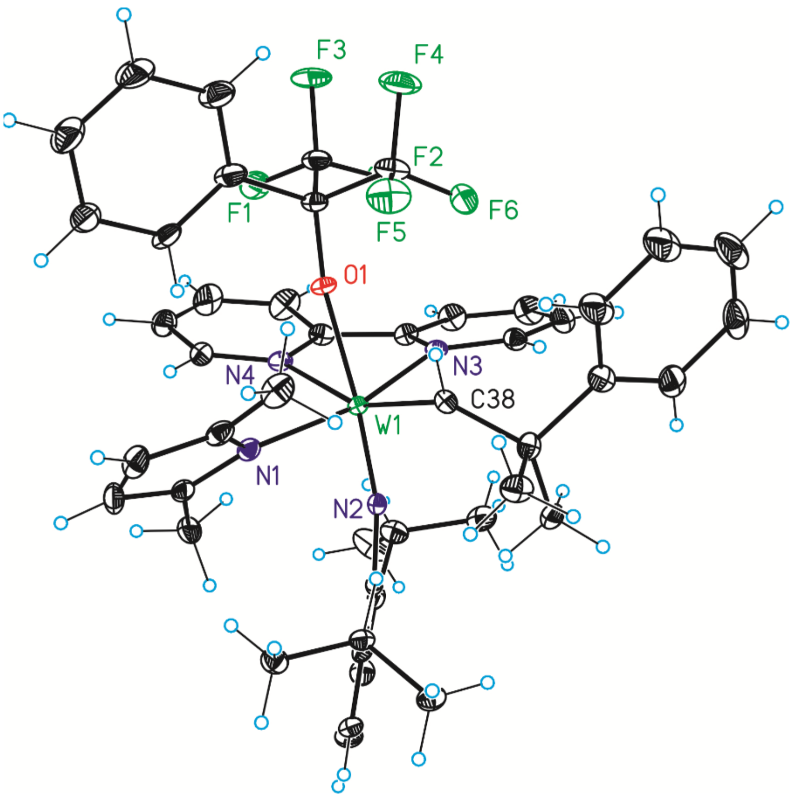

Supplementary Figure 8. Solid-state structure of W(NAr^{diiPr})(CHCMe$_2$Ph)(2,5-Me$_2$NC$_4$H$_2$)(OCPh(CF$_3$)$_2$)(2,2'-bipyridine) (Ar^{diiPr} = 2,6-diisopropyl-phenyl; **9**) determined by single-crystal X-ray diffraction; the asymmetric unit contains one molecule of the metal complex; the phenyl ring of the alkoxide ligand and one of the isopropyl groups of the imido ligand is disordered in two orientations (ratio: 60:40); the structure is of good quality with an R1 value of 3.46 %; for further structural details see the corresponding section

1.6. Synthesis of W(NAr^{diiPr})(CHCMe$_2$Ph)(2,5-Me$_2$NC$_4$H$_2$)(OCPh(CF$_3$)$_2$)(5,5'-dimethyl-2,2'-bipyridine) (Ar^{diiPr} = 2,6-diisopropyl-phenyl; 10)

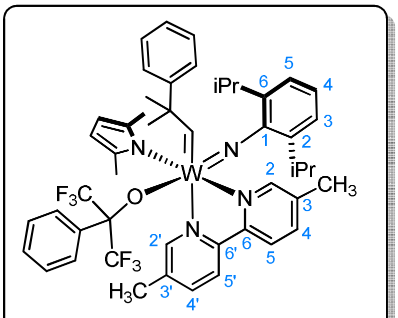

10

The bispyrrolide precursor W(CHCMe$_2$Ph)(NAr^{diiPr})(Me$_2$Pyr)$_2$ (Ar^{diiPr} = 2,6-diisopropylphenyl, Me$_2$Pyr = 2,5-dimethylpyrrolide) (347 mg, 0.51 mmol) was dissolved in benzene (5 mL). Ph(CF$_3$)$_2$COH (86 microL, 0.51 mmol) was added, and the reaction mixture was stirred for an hour at room temperature. An aliquot of the reaction mixture was analyzed by ^{1}H NMR and found to be of a purity of >98%. 5,5'-Dimethyl-2,2'-bipyridine (94 mg, 0.51 mmol was added. The reaction mixture turned red immediately. The rest of the bipyridine derivative was rinsed into the reaction mixture with benzene (1 mL). The reaction mixture was stirred for 1 hour at room temperature, and then, without checking it by NMR, it was concentrated to about 1 mL. In the course of the procedure, a considerable amount of product precipitated from the solution. Pentane (about 6 mL) was added, which resulted in a large amount of orange precipitate. The reaction mixture was left in the freezer for an hour. The product was isolated by filtration and dried on the frit in a vacuum-induced N$_2$ stream. Peach colored solid. Yield: 474 mg (92%).

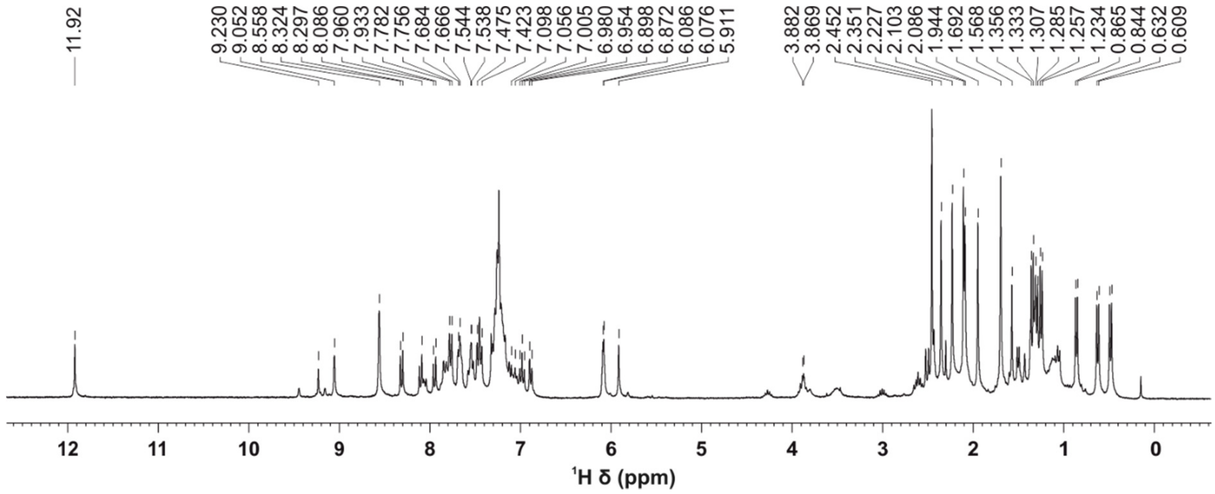

Supplementary Figure 9. ^{1}H NMR spectrum of **10** in $CDCl_3$ (c = 0.03 M) at 298 K

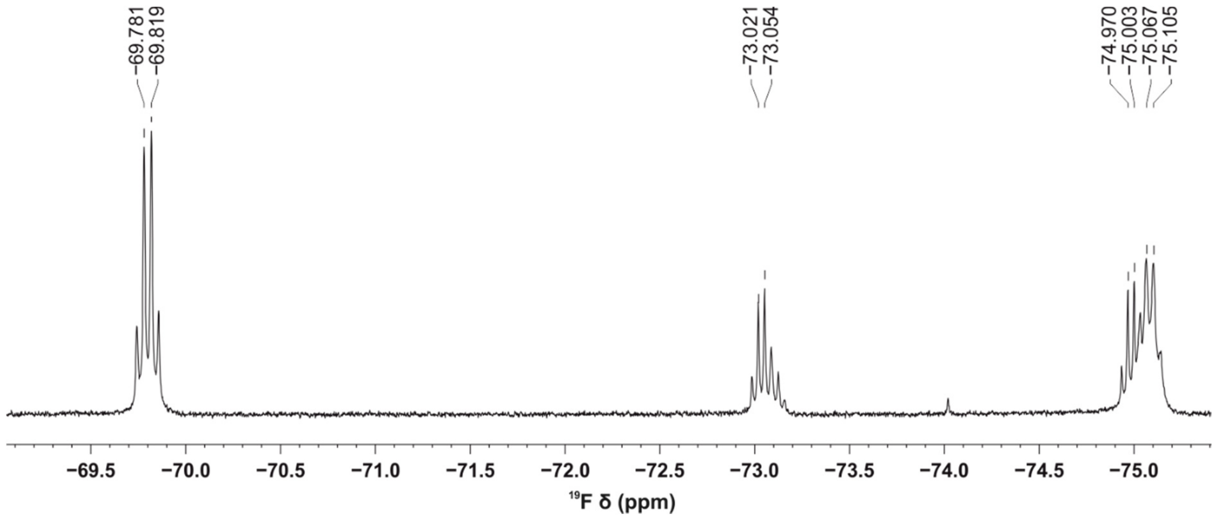

Supplementary Figure 10. ^{19}F NMR spectrum of **10** in $CDCl_3$ (c = 0.03 M) at 298 K

^{1}H NMR (300 MHz, $CDCl_3$, 25°C): δ 0.48 (d, $^{3}J_{H\text{-}H}$ = 6.9 Hz, 3H, NAr 2-iPr $CH_3$1); δ 0.63 (d, 3H, $^{3}J_{H\text{-}H}$ = 6.9 Hz, NAr 2-iPr $CH_3$2); 0.86 (d, $^{3}J_{H\text{-}H}$ = 6.6 Hz, 3H, NAr 6-iPr $CH_3$1); 1.30 (d, $^{3}J_{H\text{-}H}$ = 6.6 Hz, 3H, NAr 6-iPr $CH_3$2); 1.69 (s, 3H, neoph $CH_3$1); 1.95 (s, 3H, Me_2Pyr 2-CH_3); 2.09 (s, 3H, neoph $CH_3$2); 2.11 (s, 3H, Me_2bipy 3-CH_3); 2.36 (s, 3H, Me_2Pyr 5-CH_3); 2.44 (s, 3H, Me_2bipy 3'-CH_3); 2.61 (septet, $^{3}J_{H\text{-}H}$ = 6.9 Hz, 1H, NAr 2-iPr CH); 3.89 (septet, $^{3}J_{H\text{-}H}$ = 6.6 Hz, 1H, NAr 6-iPr CH); 6.07 (AB d, 1H, Me_2Pyr 4-CH); 6.89 (dd, $^{3}J_{H\text{-}H}$ = 7.5, $^{4}J_{H\text{-}H}$ = 1.2 Hz, 1H, NAr 3); 6.10 (AB d, 1H, Me_2Pyr 3-CH); 6.99 (t, $^{3}J_{H\text{-}H}$ = 7.5 Hz, 1H, NAr 4); 7.14-7.35 (overlapped, 3H, $OC(CF_3)_2Ph$); 7.20 (dd, $^{3}J_{H\text{-}H}$ = 7.5, $^{4}J_{H\text{-}H}$ = 1.2 Hz, 1H, NAr 5); 7.29 (t, $^{3}J_{H\text{-}H}$ = 7.9 Hz, 1H, neoph para); 7.46 (t, $^{3}J_{H\text{-}H}$ = 7.9 Hz, 2H, neoph meta); 7.68 (d, $^{3}J_{H\text{-}H}$ = 7.91 Hz, 1H, Me_2bipy

4’); 7.77 (d, $^3J_{H-H}$ = 7.9 Hz, 2H, neoph ortho); 7.84 (d, $^3J_{H-H}$ = 8.2 Hz, 1H, Me_2bipy 4); 8.11 (d, $^3J_{H-H}$ = 8.2 Hz, 1H, Me_2bipy 5); 8.32 (d, $^3J_{H-H}$ = 7.9 Hz, 1H, Me_2bipy 5’); 8.56 (s, 2H, Me_2bipy 2, 2’); 11.92 (t, $^2J_{W-H}$ = 9.8 Hz, 1H, W=CH) ppm.

^{19}F NMR (282.4 MHz, $CDCl_3$, 25°C): δ -75.0 (q, $^4J_{F-F}$ = 10.8 Hz, 3F, $CF_3$1); -69.8 (q, $^4J_{F-F}$ = 10.8 Hz, 3F, $CF_3$2) ppm.

^{13}C NMR (75.5 MHz, $CDCl_3$, 25°C): δ 18.0 (Me_2bipy 3-CH_3); 18.6 (Me_2bipy 3’-CH_3); 20.6 (Me_2Pyr 2-CH_3); 20.8 (Me_2Pyr 5-CH_3); 23.7 (NAr 2-iPr CH_3); 24.1 (NAr 6-iPr $CH_3$1); 27.0 (NAr 6-iPr $CH_3$2); 27.4 (NAr 6-iPr CH); 27.7 (NAr 2-iPr CH); 30.3 (neoph $CH_3$2); 36.7 (neoph $CH_3$1); 54.1 (neoph C); 106.9 (Me_2Pyr 4-CH); 110.3 (Me_2Pyr 3-CH); 120.2 (Me_2bipy 5’); 120.6 (Me_2bipy 5); 122.0 (NAr 3); 124.6 (NAr 5); 125.2 (NAr 4); 125.9 (neoph para); 126.9 (neoph ortho); 128.3 (neoph meta); 133.2 (Me_2Pyr 2-C); 135.7 (Me_2bipy 3’); 136.1 (Me_2bipy 3); 137.6 (Me_2Pyr 5-C); 139.0 (Me_2bipy 4’); 140.3 (Me_2bipy 4); 145.8 (NAr 2); 149.9 (Me_2bipy 2, 2’); 150.2 (NAr 6); 151.2 (NAr 1); 153.7 (neoph ipso); 154.1 (bipy 6); 154.2 (bipy 6’); 285.3 (W=CH) ppm.

HRMS (ESI^+): m/z calculated for $[M – OC(CF_3)_2Ph]^+$: 769.3467, found: 769.3462; calculated for $[M – (2,5\text{-}Me_2NC_4H_2)]^+$: 918.3054, found: 918.3036.

1.7. Synthesis of Mo(NAr^{diiPr})($CHCMe_2Ph$)($OCMe(CF_3)_2$)$_2$(4,4’-dibromo-2,2’-bipyridine) (Ar^{diiPr} = 2,6-diisopropyl-phenyl; 11)

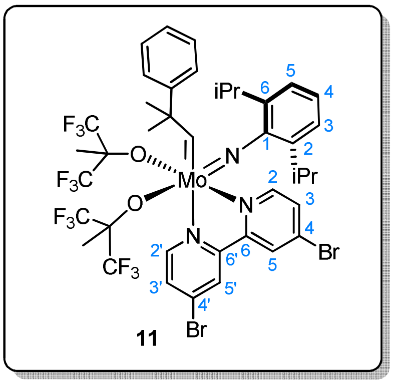

The bispyrrolide precursor Mo($CHCMe_2Ph$)(NAr^{diiPr})(Me_2Pyr)$_2$ (Ar^{diiPr} = 2,6-diisopropylphenyl, Me_2Pyr = 2,5-dimethylpyrrolide) (160 mg, 0.27 mmol) was dissolved in toluene (2 mL). $Me(CF_3)_2COH$ (73 microL, 0.594 mmol) was added, and the reaction mixture was stirred

for two hours at room temperature. NMR analyses of an aliquot confirmed conversion into the 14-electron bisalkoxide complex. 4,4'-Dibromo-2,2'-bipyridine (85 mg, 0.27 mmol) dissolved in toluene (1 mL) was added to the reaction mixture. The residues of the N-donor were rinsed into the reaction mixture with one more milliliter of toluene. The toluene was evaporated in vacuum, and the remaining oily solids were triturated in pentane. The product crystallized from the mixture. Yellow crystals. Yield: 230 mg (79%).

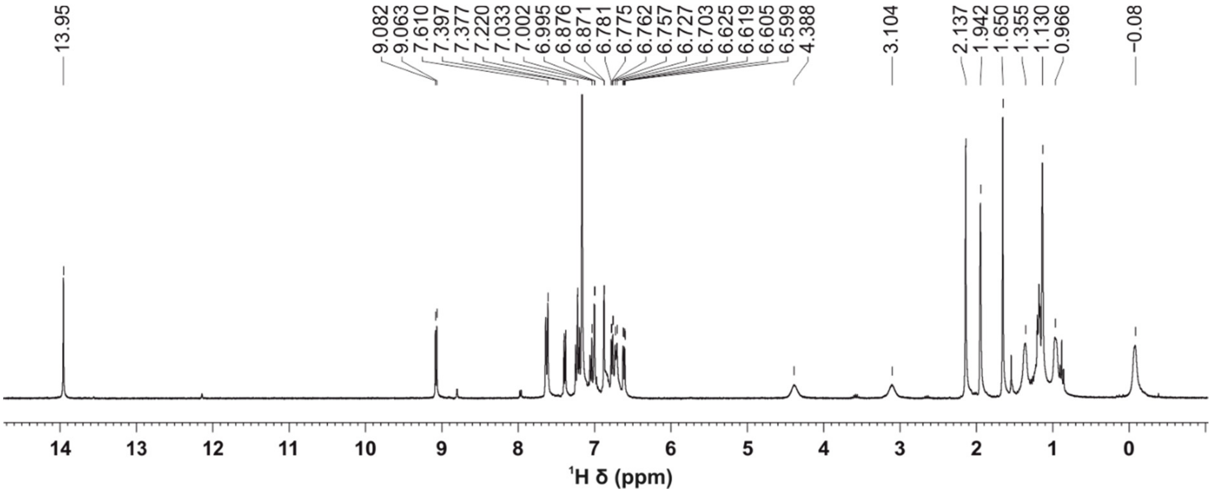

Supplementary Figure 11. ^{1}H NMR spectrum of **11** in C_6D_6 (c = 0.05 M) at 298 K

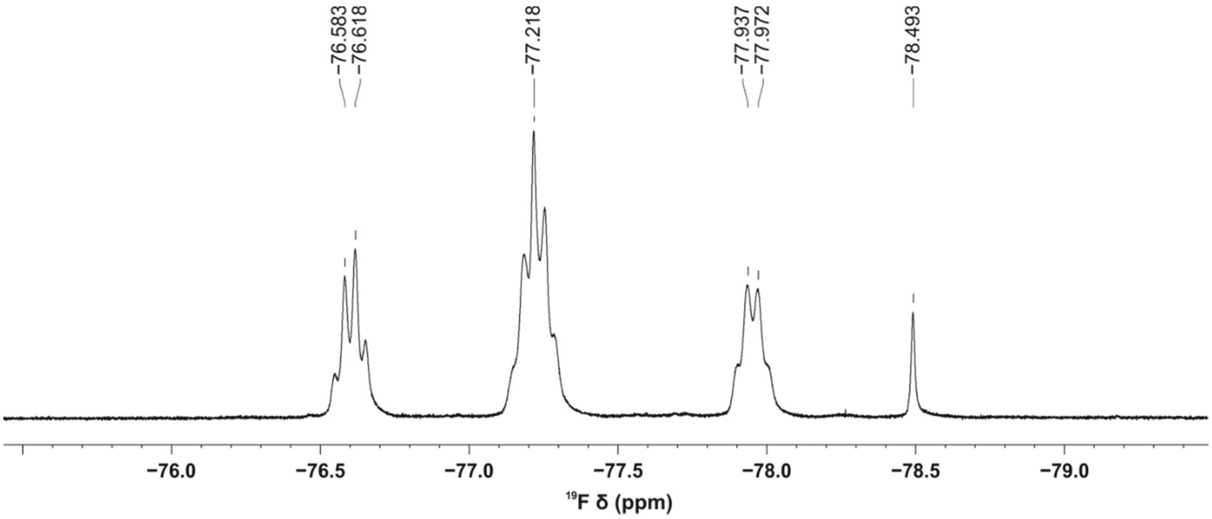

Supplementary Figure 12. ^{19}F NMR spectrum of **11** in C_6D_6 (c = 0.05 M) at 298 K

^{1}H-NMR (300 MHz, C_6D_6, 25°C): δ -0.08 (br, 3H, NAr 2-iPr $CH_3$1), 0.96 (br, 3H, NAr 2-iPr $CH_3$2), 1.15 (br, 3H, $OC(CF_3)_2CH_3$1), 1.18 (d, 3H, NAr 6-iPr $CH_3$1), 1.36 (br, 3H, NAr 6-iPr $CH_3$2), 1.65 (s, 3H, neoph $CH_3$1), 1.94 (br, 3H, $OC(CF_3)_2CH_3$2), 2.14 (s, 3H, neoph $CH_3$2), 3.10 (br, 1H, NAr 2-iPr CH), 3.57 (br, 1H,

NAr 6-iPr CH), 6.61 (dd, $^3J_{HH}$ = 6.0 Hz, $^4J_{HH}$ = 1.9 Hz, 1H, Br_2-bipy 4), 6.72 (br, 2H, NAr 3, 5), 6.76 (dd, $^3J_{HH}$ = 5.6 Hz, $^4J_{HH}$ = 1.6 Hz, 1H, Br_2-bipy 3’), 6.87 (d, $^4J_{HH}$ = 1.6 Hz, 1H, Br_2-bipy 5’), 6.99 (d, , $^4J_{HH}$ = 1.9 Hz, 1H, Br_2-bipy 5), 7.03 (m, 4H, neoph para), 7.22 (m, 2H, neoph meta), 7.39 (d, $^3J_{HH}$ = 6.0 Hz, 1H, Br_2-bipy 2), 7.62 (m, 2H, neoph ortho), 9.07 (d, $^3J_{HH}$ = 5.6 Hz, 1H, Br_2-bipy 2’), 13.95 ppm (s, 1H, Mo=CH) ppm.

^{19}F-NMR (282.4 MHz, C_6D_6, 25°C): δ -78.0 ppm (q, $J^4_{F\text{-}F}$ = 9.93 Hz, 3F, $CF_3$1); -77.3 (overlapped q, 3F, $CF_3$2); -77.2 (overlapped q, 3F, $CF_3$3); -76.6 (q, $J^4_{F\text{-}F}$ = 9.93 Hz, 3F, $CF_3$4) ppm.

^{13}C-NMR (75.5 MHz, C_6D_6, 25°C): δ 18.4 ($OC(CF_3)_2\underline{C}H_3$2), 19.0 ($OC(CF_3)_2\underline{C}H_3$1), 23.7 (NAr iPr CH_3), 28.7 (NAr iPr CH), 28.5 (neoph $CH_3$2), 31.9 (neoph $CH_3$1), 54.6 (neoph C), 80.0 ($O\underline{C}(CF_3)_2CH_3$), 80.4 ($O\underline{C}(CF_3)_2CH_3$), 125.0 ($OC\underline{C}F_3CH_3CF_3$), 126.2 (neoph para), 126.4 ($OCCF_3CH_3\underline{C}F_3$), 126.7 (neoph ortho), 128.5 (neoph meta), 129.2 (Br_2-bipy 3), 129.5 (Br_2-bipy 3’), 135.5 (Br_2-bipy 4’), 136.3 (Br_2-bipy 4), 147.2 (NAr 2), 147.9 (NAr 6), 149.5 (neoph ipso), 149.9 (Br_2-bipy 2’), 151.0 (Br_2-bipy 6’), 153.4 (NAr 1), 154.1 (Br_2-bipy 6), 159.1 (Br_2-bipy 2), 309.4 ppm (Mo=CH) ppm.

HRMS (ESI$^+$): m/z calculated for $[M – OC(CF_3)_2(CH_3)]^+$: 898.0340, found: 898.0323.

1.8. Details of the solid-state structure of 8 and 9

Supplementary Table 1. Crystal data and structure refinement for **8**

Identification code	**8**	
Empirical formula	C75 H75 N3 O2 Si2 W	
Formula weight	1290.41	
Temperature	100(2) K	
Wavelength	0.71073 Å	
Crystal system	Monoclinic	
Space group	P2(1)/c	
Unit cell dimensions	a = 13.0756(7)Å	α= 90°.
	b = 20.4377(12)Å	β = 93.3315(18)°.
	c = 23.3563(13)Å	γ = 90°.
Volume	6231.1(6) Å^3	
Z	4	
Density (calculated)	1.376 Mg/m^3	
Absorption coefficient	1.942 mm^{-1}	
F(000)	2656	
Crystal size	0.20 x 0.20 x 0.10 mm^3	

Theta range for data collection	1.325 to 32.547°.
Index ranges	-15<=h<=19,-25<=k<=30,-35<=l<=26
Reflections collected	57206
Independent reflections	19856[R(int) = 0.0406]
Completeness to theta =32.547°	87.7%
Absorption correction	Empirical
Max. and min. transmission	0.829 and 0.634
Refinement method	Full-matrix least-squares on F^2
Data / restraints / parameters	19856/ 110/ 809
Goodness-of-fit on F^2	1.018
Final R indices [I>2sigma(I)]	R1 = 0.0307, wR2 = 0.0619
R indices (all data)	R1 = 0.0603, wR2 = 0.0705
Largest diff. peak and hole	1.276 and -0.721 e.$Å^{-3}$

Supplementary Table 2. Bond lengths [Å] and angles [°] for **8**

Bond lengths----

W1-N1	1.7730(18)
W1-C1	1.932(2)
W1-O1	2.0019(15)
W1-O2	2.0176(15)
W1-N3	2.2437(18)
W1-N2	2.3817(19)
Si1-O1	1.6185(16)
Si1-C45	1.875(6)
Si1-C45'	1.879(8)
Si1-C33	1.880(2)
Si1-C39	1.881(3)
Si2-O2	1.5987(16)
Si2-C57	1.884(2)
Si2-C51	1.885(2)
Si2-C63	1.886(2)
N1-C11	1.400(3)
N2-C23	1.331(3)
N2-C27	1.345(3)
N3-C32	1.344(3)
N3-C28	1.354(3)
C1-C2	1.527(3)
C2-C9	1.533(3)
C2-C10	1.546(3)
C2-C3	1.548(3)
C3-C4	1.378(4)
C3-C8	1.392(3)
C4-C5	1.388(4)
C5-C6	1.394(4)
C6-C7	1.386(4)
C7-C8	1.389(3)
C11-C12	1.414(3)
C11-C16	1.419(3)
C12-C13	1.395(3)
C12-C17	1.512(3)
C13-C14	1.384(3)

C14-C15	1.380(3)
C15-C16	1.391(3)
C16-C20	1.515(3)
C17-C18	1.527(3)
C17-C19	1.533(4)
C20-C21	1.527(3)
C20-C22	1.531(3)
C23-C24	1.389(3)
C24-C25	1.379(4)
C25-C26	1.382(3)
C26-C27	1.391(3)
C27-C28	1.479(3)
C28-C29	1.394(3)
C29-C30	1.379(3)
C30-C31	1.383(3)
C31-C32	1.378(3)
C33-C38	1.396(3)
C33-C34	1.399(3)
C34-C35	1.389(3)
C35-C36	1.384(4)
C36-C37	1.381(4)
C37-C38	1.392(4)
C39-C40	1.396(3)
C39-C44	1.396(3)
C40-C41	1.400(4)
C41-C42	1.384(4)
C42-C43	1.377(4)
C43-C44	1.388(3)
C45-C46	1.383(7)
C45-C50	1.440(12)
C46-C47	1.408(8)
C47-C48	1.350(11)
C48-C49	1.368(8)
C49-C50	1.390(7)
C45'-C46'	1.378(6)
C45'-C50'	1.393(6)
C46'-C47'	1.389(6)
C47'-C48'	1.397(6)

C48'-C49'	1.387(6)
C49'-C50'	1.390(5)
C51-C56	1.395(3)
C51-C52	1.397(3)
C52-C53	1.394(3)
C53-C54	1.384(3)
C54-C55	1.386(3)
C55-C56	1.390(3)
C57-C58	1.394(3)
C57-C62	1.397(3)
C58-C59	1.391(3)
C59-C60	1.378(4)
C60-C61	1.392(4)
C61-C62	1.390(3)
C63-C68	1.397(3)
C63-C64	1.405(3)
C64-C65	1.391(3)
C65-C66	1.379(3)
C66-C67	1.384(3)
C67-C68	1.394(3)
C1S-C2S	1.384(4)
C1S-C6S	1.387(4)
C1S-C7S	1.500(4)
C2S-C3S	1.388(4)
C3S-C4S	1.386(4)
C4S-C5S	1.379(4)
C5S-C6S	1.386(4)

Angles----------

N1-W1-C1	101.21(10)
N1-W1-O1	99.32(7)
C1-W1-O1	102.14(8)
N1-W1-O2	164.18(7)
C1-W1-O2	91.64(9)
O1-W1-O2	86.79(6)
N1-W1-N3	91.30(7)
C1-W1-N3	97.27(8)
O1-W1-N3	155.52(7)

O2-W1-N3	77.83(6)
N1-W1-N2	86.75(7)
C1-W1-N2	165.75(9)
O1-W1-N2	88.02(6)
O2-W1-N2	78.84(7)
N3-W1-N2	70.52(7)
O1-Si1-C45	112.7(4)
O1-Si1-C45'	112.2(5)
O1-Si1-C33	109.79(10)
C45-Si1-C33	107.2(5)
C45'-Si1-C33	106.7(8)
O1-Si1-C39	113.52(10)
C45-Si1-C39	107.1(7)
C45'-Si1-C39	108.2(11)
C33-Si1-C39	106.15(11)
O2-Si2-C57	108.38(10)
O2-Si2-C51	109.47(10)
C57-Si2-C51	110.18(10)
O2-Si2-C63	113.90(9)
C57-Si2-C63	105.99(10)
C51-Si2-C63	108.85(11)
Si1-O1-W1	139.42(10)
Si2-O2-W1	166.08(10)
C11-N1-W1	170.75(16)
C23-N2-C27	118.7(2)
C23-N2-W1	124.80(16)
C27-N2-W1	116.09(15)
C32-N3-C28	118.1(2)
C32-N3-W1	121.63(16)
C28-N3-W1	120.12(15)
C2-C1-W1	148.23(19)
C1-C2-C9	109.2(2)
C1-C2-C10	113.3(2)
C9-C2-C10	107.0(2)
C1-C2-C3	105.37(19)
C9-C2-C3	111.2(2)
C10-C2-C3	110.9(2)
C4-C3-C8	117.8(2)

C4-C3-C2	121.0(2)
C8-C3-C2	121.0(2)
C3-C4-C5	121.0(3)
C4-C5-C6	120.6(3)
C7-C6-C5	119.0(3)
C6-C7-C8	119.4(3)
C7-C8-C3	122.0(2)
N1-C11-C12	119.6(2)
N1-C11-C16	120.4(2)
C12-C11-C16	120.0(2)
C13-C12-C11	118.4(2)
C13-C12-C17	118.5(2)
C11-C12-C17	123.0(2)
C14-C13-C12	121.5(2)
C15-C14-C13	119.9(2)
C14-C15-C16	121.2(2)
C15-C16-C11	118.9(2)
C15-C16-C20	118.9(2)
C11-C16-C20	122.1(2)
C12-C17-C18	113.2(2)
C12-C17-C19	110.8(2)
C18-C17-C19	108.4(2)
C16-C20-C21	110.0(2)
C16-C20-C22	112.76(19)
C21-C20-C22	109.8(2)
N2-C23-C24	123.0(2)
C25-C24-C23	118.4(2)
C24-C25-C26	119.0(2)
C25-C26-C27	119.5(2)
N2-C27-C26	121.3(2)
N2-C27-C28	115.3(2)
C26-C27-C28	123.4(2)
N3-C28-C29	120.8(2)
N3-C28-C27	116.9(2)
C29-C28-C27	122.3(2)
C30-C29-C28	120.6(2)
C29-C30-C31	118.0(2)
C32-C31-C30	119.1(2)

N3-C32-C31	123.3(2)
C38-C33-C34	116.8(2)
C38-C33-Si1	121.39(19)
C34-C33-Si1	121.64(18)
C35-C34-C33	121.5(2)
C36-C35-C34	120.4(3)
C37-C36-C35	119.2(3)
C36-C37-C38	120.2(3)
C37-C38-C33	121.8(3)
C40-C39-C44	116.7(2)
C40-C39-Si1	121.7(2)
C44-C39-Si1	121.37(18)
C39-C40-C41	121.8(3)
C42-C41-C40	119.5(3)
C43-C42-C41	120.1(3)
C42-C43-C44	119.7(3)
C43-C44-C39	122.2(2)
C46-C45-C50	114.3(8)
C46-C45-Si1	122.6(8)
C50-C45-Si1	122.3(7)
C45-C46-C47	124.2(6)
C48-C47-C46	118.1(8)
C47-C48-C49	120.7(6)
C48-C49-C50	121.6(5)
C49-C50-C45	119.9(5)
C46'-C45'-C50'	117.2(8)
C46'-C45'-Si1	117.2(12)
C50'-C45'-Si1	120.8(8)
C45'-C46'-C47'	120.4(7)
C46'-C47'-C48'	120.4(7)
C49'-C48'-C47'	119.0(6)
C48'-C49'-C50'	119.3(6)
C49'-C50'-C45'	121.6(5)
C56-C51-C52	117.2(2)
C56-C51-Si2	123.08(18)
C52-C51-Si2	119.71(17)
C53-C52-C51	121.9(2)
C54-C53-C52	119.5(2)

C53-C54-C55	119.8(2)
C54-C55-C56	120.2(2)
C55-C56-C51	121.4(2)
C58-C57-C62	117.2(2)
C58-C57-Si2	123.35(18)
C62-C57-Si2	119.45(18)
C59-C58-C57	121.4(2)
C60-C59-C58	120.6(2)
C59-C60-C61	119.3(2)
C62-C61-C60	119.8(2)
C61-C62-C57	121.8(2)
C68-C63-C64	116.9(2)
C68-C63-Si2	122.76(18)
C64-C63-Si2	120.35(18)
C65-C64-C63	121.5(2)
C66-C65-C64	120.4(2)
C65-C66-C67	119.4(2)
C66-C67-C68	120.3(2)
C67-C68-C63	121.5(2)
C2S-C1S-C6S	117.7(3)
C2S-C1S-C7S	121.0(3)
C6S-C1S-C7S	121.3(3)
C1S-C2S-C3S	121.5(3)
C4S-C3S-C2S	119.7(3)
C5S-C4S-C3S	119.7(3)
C4S-C5S-C6S	119.7(3)
C5S-C6S-C1S	121.6(3)

Supplementary Table 3. Torsion angles [°] for **8**

C45-Si1-O1-W1	74.3(8)
C45'-Si1-O1-W1	75.3(12)
C33-Si1-O1-W1	-166.29(13)
C39-Si1-O1-W1	-47.68(17)
C57-Si2-O2-W1	150.9(4)
C51-Si2-O2-W1	-88.9(4)
C63-Si2-O2-W1	33.2(5)
W1-C1-C2-C9	132.4(3)
W1-C1-C2-C10	13.3(4)
W1-C1-C2-C3	-108.1(3)
C1-C2-C3-C4	-71.7(3)
C9-C2-C3-C4	46.4(3)
C10-C2-C3-C4	165.3(2)
C1-C2-C3-C8	103.0(3)
C9-C2-C3-C8	-138.8(2)
C10-C2-C3-C8	-19.9(3)
C8-C3-C4-C5	-2.6(4)
C2-C3-C4-C5	172.4(2)
C3-C4-C5-C6	-0.3(4)
C4-C5-C6-C7	2.6(4)
C5-C6-C7-C8	-1.9(4)
C6-C7-C8-C3	-1.1(4)
C4-C3-C8-C7	3.3(4)
C2-C3-C8-C7	-171.6(2)
N1-C11-C12-C13	178.4(2)
C16-C11-C12-C13	-0.6(3)
N1-C11-C12-C17	-4.0(3)
C16-C11-C12-C17	176.9(2)
C11-C12-C13-C14	0.6(4)
C17-C12-C13-C14	-177.1(2)
C12-C13-C14-C15	0.2(4)
C13-C14-C15-C16	-0.9(4)
C14-C15-C16-C11	0.8(4)
C14-C15-C16-C20	178.3(2)
N1-C11-C16-C15	-179.1(2)
C12-C11-C16-C15	0.0(3)

681	N1-C11-C16-C20	3.5(3)
682	C12-C11-C16-C20	-177.5(2)
683	C13-C12-C17-C18	-54.9(3)
684	C11-C12-C17-C18	127.6(2)
685	C13-C12-C17-C19	67.1(3)
686	C11-C12-C17-C19	-110.5(3)
687	C15-C16-C20-C21	-73.3(3)
688	C11-C16-C20-C21	104.1(3)
689	C15-C16-C20-C22	49.6(3)
690	C11-C16-C20-C22	-132.9(2)
691	C27-N2-C23-C24	-2.1(4)
692	W1-N2-C23-C24	170.39(18)
693	N2-C23-C24-C25	-0.3(4)
694	C23-C24-C25-C26	1.8(4)
695	C24-C25-C26-C27	-0.9(4)
696	C23-N2-C27-C26	3.1(3)
697	W1-N2-C27-C26	-170.09(17)
698	C23-N2-C27-C28	-174.9(2)
699	W1-N2-C27-C28	12.0(2)
700	C25-C26-C27-N2	-1.6(4)
701	C25-C26-C27-C28	176.2(2)
702	C32-N3-C28-C29	-2.2(3)
703	W1-N3-C28-C29	-177.40(16)
704	C32-N3-C28-C27	175.4(2)
705	W1-N3-C28-C27	0.3(3)
706	N2-C27-C28-N3	-8.4(3)
707	C26-C27-C28-N3	173.7(2)
708	N2-C27-C28-C29	169.2(2)
709	C26-C27-C28-C29	-8.7(4)
710	N3-C28-C29-C30	0.4(4)
711	C27-C28-C29-C30	-177.1(2)
712	C28-C29-C30-C31	1.4(4)
713	C29-C30-C31-C32	-1.4(4)
714	C28-N3-C32-C31	2.3(3)
715	W1-N3-C32-C31	177.38(18)
716	C30-C31-C32-N3	-0.5(4)
717	O1-Si1-C33-C38	142.7(2)
718	C45-Si1-C33-C38	-94.6(7)

C45'-Si1-C33-C38	-95.5(10)
C39-Si1-C33-C38	19.7(2)
O1-Si1-C33-C34	-41.6(2)
C45-Si1-C33-C34	81.1(7)
C45'-Si1-C33-C34	80.2(10)
C39-Si1-C33-C34	-164.67(19)
C38-C33-C34-C35	-1.0(4)
Si1-C33-C34-C35	-176.87(19)
C33-C34-C35-C36	1.0(4)
C34-C35-C36-C37	0.3(4)
C35-C36-C37-C38	-1.5(4)
C36-C37-C38-C33	1.4(5)
C34-C33-C38-C37	-0.2(4)
Si1-C33-C38-C37	175.7(2)
O1-Si1-C39-C40	144.40(19)
C45-Si1-C39-C40	19.3(4)
C45'-Si1-C39-C40	19.2(5)
C33-Si1-C39-C40	-94.9(2)
O1-Si1-C39-C44	-41.5(2)
C45-Si1-C39-C44	-166.5(4)
C45'-Si1-C39-C44	-166.6(5)
C33-Si1-C39-C44	79.2(2)
C44-C39-C40-C41	-1.7(4)
Si1-C39-C40-C41	172.7(2)
C39-C40-C41-C42	0.5(4)
C40-C41-C42-C43	1.3(4)
C41-C42-C43-C44	-2.0(4)
C42-C43-C44-C39	0.8(4)
C40-C39-C44-C43	1.0(4)
Si1-C39-C44-C43	-173.42(19)
O1-Si1-C45-C46	-38(2)
C33-Si1-C45-C46	-158.7(18)
C39-Si1-C45-C46	87.7(19)
O1-Si1-C45-C50	131.7(12)
C33-Si1-C45-C50	10.8(17)
C39-Si1-C45-C50	-102.8(15)
C50-C45-C46-C47	13(3)
Si1-C45-C46-C47	-176.4(19)

C45-C46-C47-C48	-10(3)
C46-C47-C48-C49	2(2)
C47-C48-C49-C50	0.9(15)
C48-C49-C50-C45	3.4(13)
C46-C45-C50-C49	-10(2)
Si1-C45-C50-C49	179.7(9)
O1-Si1-C45'-C46'	-40(3)
C33-Si1-C45'-C46'	-160(2)
C39-Si1-C45'-C46'	86(3)
O1-Si1-C45'-C50'	165(2)
C33-Si1-C45'-C50'	45(3)
C39-Si1-C45'-C50'	-69(3)
C50'-C45'-C46'-C47'	-17(5)
Si1-C45'-C46'-C47'	-173(3)
C45'-C46'-C47'-C48'	10(4)
C46'-C47'-C48'-C49'	-1(3)
C47'-C48'-C49'-C50'	-1(2)
C48'-C49'-C50'-C45'	-7(2)
C46'-C45'-C50'-C49'	16(4)
Si1-C45'-C50'-C49'	170.6(14)
O2-Si2-C51-C56	167.61(19)
C57-Si2-C51-C56	-73.3(2)
C63-Si2-C51-C56	42.5(2)
O2-Si2-C51-C52	-15.0(2)
C57-Si2-C51-C52	104.09(19)
C63-Si2-C51-C52	-140.08(18)
C56-C51-C52-C53	1.0(3)
Si2-C51-C52-C53	-176.57(18)
C51-C52-C53-C54	-0.8(4)
C52-C53-C54-C55	0.4(4)
C53-C54-C55-C56	-0.1(4)
C54-C55-C56-C51	0.2(4)
C52-C51-C56-C55	-0.7(3)
Si2-C51-C56-C55	176.79(18)
O2-Si2-C57-C58	147.49(19)
C51-Si2-C57-C58	27.7(2)
C63-Si2-C57-C58	-89.9(2)
O2-Si2-C57-C62	-34.4(2)

C51-Si2-C57-C62	-154.21(18)
C63-Si2-C57-C62	88.2(2)
C62-C57-C58-C59	-0.5(4)
Si2-C57-C58-C59	177.58(19)
C57-C58-C59-C60	0.6(4)
C58-C59-C60-C61	-0.1(4)
C59-C60-C61-C62	-0.4(4)
C60-C61-C62-C57	0.4(4)
C58-C57-C62-C61	0.0(4)
Si2-C57-C62-C61	-178.15(19)
O2-Si2-C63-C68	-94.3(2)
C57-Si2-C63-C68	146.62(19)
C51-Si2-C63-C68	28.1(2)
O2-Si2-C63-C64	86.1(2)
C57-Si2-C63-C64	-33.0(2)
C51-Si2-C63-C64	-151.47(19)
C68-C63-C64-C65	0.5(4)
Si2-C63-C64-C65	-179.84(19)
C63-C64-C65-C66	-0.2(4)
C64-C65-C66-C67	-0.2(4)
C65-C66-C67-C68	0.2(4)
C66-C67-C68-C63	0.2(4)
C64-C63-C68-C67	-0.5(3)
Si2-C63-C68-C67	179.85(18)
C6S-C1S-C2S-C3S	0.1(4)
C7S-C1S-C2S-C3S	-179.5(3)
C1S-C2S-C3S-C4S	-0.6(4)
C2S-C3S-C4S-C5S	0.8(4)
C3S-C4S-C5S-C6S	-0.7(4)
C4S-C5S-C6S-C1S	0.2(4)
C2S-C1S-C6S-C5S	0.1(4)
C7S-C1S-C6S-C5S	179.6(3)

Supplementary Table 4. Crystal data and structure refinement for **9**

Identification code	**9**	
Empirical formula	C47 H50 F6 N4 O W	
Formula weight	984.76	
Temperature	100(2) K	
Wavelength	0.71073 Å	
Crystal system	Monoclinic	
Space group	P2(1)/c	
Unit cell dimensions	a = 14.0695(6)Å	α = 90°.
	b = 13.6259(5)Å	β = 95.9850(14)°.
	c = 22.1224(9)Å	γ = 90°.
Volume	4218.0(3) $Å^3$	
Z	4	
Density (calculated)	1.551 Mg/m^3	
Absorption coefficient	2.806 mm^{-1}	
F(000)	1984	
Crystal size	0.20 x 0.12 x 0.05 mm^3	
Theta range for data collection	1.758 to 32.603°.	
Index ranges	-21<=h<=21,-20<=k<=20,-32<=l<=17	
Reflections collected	54907	
Independent reflections	14174[R(int) = 0.0584]	
Completeness to theta =32.603°	91.9%	
Absorption correction	Multi-scan	
Max. and min. transmission	0.872 and 0.758	
Refinement method	Full-matrix least-squares on F^2	
Data / restraints / parameters	14174/ 344/ 614	
Goodness-of-fit on F^2	1.021	
Final R indices [I>2sigma(I)]	R1 = 0.0346, wR2 = 0.0589	
R indices (all data)	R1 = 0.0595, wR2 = 0.0654	
Largest diff. peak and hole	2.380 and -1.139 e.$Å^{-3}$	

Supplementary Table 5. Bond lengths [Å] and angles [°] for **9**

Bond lengths----

W1-N2	1.756(2)
W1-C38	1.941(2)
W1-O1	2.0551(17)
W1-N1	2.127(2)
W1-N3	2.244(2)
W1-N4	2.385(2)
N1-C5	1.401(3)
N1-C2	1.402(3)
N2-C7	1.399(3)
N3-C28	1.342(3)
N3-C24	1.357(3)
N4-C23	1.339(3)
N4-C19	1.341(3)
O1-C29	1.359(3)
C1-C2	1.493(4)
C2-C3	1.360(4)
C3-C4	1.415(4)
C4-C5	1.366(4)
C5-C6	1.507(4)
C7-C8	1.415(4)
C7-C12	1.421(3)
C8-C9	1.387(4)
C8-C13	1.530(3)
C9-C10	1.386(4)
C10-C11	1.387(4)
C11-C12	1.385(4)
C12-C16	1.516(4)
C13-C15	1.520(4)
C13-C14	1.542(4)
C16-C18'	1.495(7)
C16-C17	1.518(5)
C16-C18	1.551(5)
C16-C17'	1.553(6)
C19-C20	1.383(4)
C20-C21	1.379(4)

903	C21-C22	1.365(4)
904	C22-C23	1.402(4)
905	C23-C24	1.475(4)
906	C24-C25	1.387(4)
907	C25-C26	1.376(4)
908	C26-C27	1.382(4)
909	C27-C28	1.373(4)
910	C29-C32	1.528(6)
911	C29-C32'	1.543(7)
912	C29-C31	1.551(4)
913	C29-C30	1.552(4)
914	C30-F2	1.339(3)
915	C30-F1	1.340(3)
916	C30-F3	1.345(3)
917	C31-F6	1.330(3)
918	C31-F5	1.344(3)
919	C31-F4	1.346(3)
920	C32-C37	1.386(5)
921	C32-C33	1.408(4)
922	C33-C34	1.377(5)
923	C34-C35	1.384(6)
924	C35-C36	1.385(5)
925	C36-C37	1.386(5)
926	C32'-C37'	1.387(5)
927	C32'-C33'	1.409(5)
928	C33'-C34'	1.378(6)
929	C34'-C35'	1.385(6)
930	C35'-C36'	1.386(6)
931	C36'-C37'	1.386(6)
932	C38-C39	1.534(4)
933	C39-C41	1.534(4)
934	C39-C42	1.545(4)
935	C39-C40	1.553(4)
936	C42-C47	1.384(4)
937	C42-C43	1.398(4)
938	C43-C44	1.384(4)
939	C44-C45	1.361(5)
940	C45-C46	1.377(5)

C46-C47 1.399(4)

Angles----------

N2-W1-C38	97.69(10)
N2-W1-O1	172.45(8)
C38-W1-O1	89.82(9)
N2-W1-N1	90.14(9)
C38-W1-N1	104.76(9)
O1-W1-N1	88.67(8)
N2-W1-N3	93.38(9)
C38-W1-N3	92.47(9)
O1-W1-N3	85.50(7)
N1-W1-N3	161.80(8)
N2-W1-N4	97.77(8)
C38-W1-N4	157.43(9)
O1-W1-N4	74.82(7)
N1-W1-N4	91.56(8)
N3-W1-N4	70.28(8)
C5-N1-C2	105.6(2)
C5-N1-W1	125.94(17)
C2-N1-W1	127.91(18)
C7-N2-W1	167.15(19)
C28-N3-C24	117.5(2)
C28-N3-W1	122.10(17)
C24-N3-W1	119.91(18)
C23-N4-C19	119.0(2)
C23-N4-W1	115.83(18)
C19-N4-W1	124.66(17)
C29-O1-W1	170.11(17)
C3-C2-N1	109.8(2)
C3-C2-C1	127.7(3)
N1-C2-C1	122.5(3)
C2-C3-C4	107.5(3)
C5-C4-C3	107.3(3)
C4-C5-N1	109.7(2)
C4-C5-C6	124.3(3)
N1-C5-C6	125.5(2)
N2-C7-C8	120.2(2)

979	N2-C7-C12	118.7(2)
980	C8-C7-C12	120.9(2)
981	C9-C8-C7	118.0(2)
982	C9-C8-C13	119.3(2)
983	C7-C8-C13	122.7(2)
984	C10-C9-C8	121.6(3)
985	C9-C10-C11	119.7(3)
986	C12-C11-C10	121.4(2)
987	C11-C12-C7	118.2(2)
988	C11-C12-C16	122.7(2)
989	C7-C12-C16	119.1(2)
990	C15-C13-C8	113.1(2)
991	C15-C13-C14	108.6(2)
992	C8-C13-C14	110.7(2)
993	C18'-C16-C12	112.6(4)
994	C12-C16-C17	115.6(3)
995	C12-C16-C18	107.2(3)
996	C17-C16-C18	108.6(3)
997	C18'-C16-C17'	110.5(4)
998	C12-C16-C17'	110.7(4)
999	N4-C19-C20	122.6(3)
1000	C21-C20-C19	118.6(3)
1001	C22-C21-C20	119.1(3)
1002	C21-C22-C23	119.9(3)
1003	N4-C23-C22	120.7(3)
1004	N4-C23-C24	116.5(2)
1005	C22-C23-C24	122.6(3)
1006	N3-C24-C25	121.5(3)
1007	N3-C24-C23	115.5(2)
1008	C25-C24-C23	122.9(2)
1009	C26-C25-C24	119.9(3)
1010	C25-C26-C27	118.6(3)
1011	C28-C27-C26	118.8(3)
1012	N3-C28-C27	123.5(3)
1013	O1-C29-C32	114.2(4)
1014	O1-C29-C32'	111.2(6)
1015	O1-C29-C31	107.8(2)
1016	C32-C29-C31	110.9(3)

1017	C32'-C29-C31	112.6(4)
1018	O1-C29-C30	109.2(2)
1019	C32-C29-C30	106.6(3)
1020	C32'-C29-C30	107.9(4)
1021	C31-C29-C30	108.1(2)
1022	F2-C30-F1	106.5(2)
1023	F2-C30-F3	106.4(2)
1024	F1-C30-F3	105.9(2)
1025	F2-C30-C29	113.2(2)
1026	F1-C30-C29	110.5(2)
1027	F3-C30-C29	113.8(2)
1028	F6-C31-F5	107.0(2)
1029	F6-C31-F4	106.2(2)
1030	F5-C31-F4	106.5(2)
1031	F6-C31-C29	111.9(2)
1032	F5-C31-C29	110.2(2)
1033	F4-C31-C29	114.6(2)
1034	C37-C32-C33	118.0(4)
1035	C37-C32-C29	117.8(6)
1036	C33-C32-C29	123.6(6)
1037	C34-C33-C32	120.1(4)
1038	C33-C34-C35	121.1(4)
1039	C34-C35-C36	119.1(4)
1040	C35-C36-C37	120.2(4)
1041	C32-C37-C36	121.1(4)
1042	C37'-C32'-C33'	117.5(5)
1043	C37'-C32'-C29	119.2(9)
1044	C33'-C32'-C29	120.8(9)
1045	C34'-C33'-C32'	120.2(5)
1046	C33'-C34'-C35'	120.9(5)
1047	C34'-C35'-C36'	118.9(5)
1048	C35'-C36'-C37'	120.2(5)
1049	C36'-C37'-C32'	121.3(5)
1050	C39-C38-W1	143.01(19)
1051	C38-C39-C41	112.3(2)
1052	C38-C39-C42	109.1(2)
1053	C41-C39-C42	111.5(2)
1054	C38-C39-C40	108.1(2)

C41-C39-C40	108.2(2)
C42-C39-C40	107.5(2)
C47-C42-C43	117.3(3)
C47-C42-C39	123.1(3)
C43-C42-C39	119.6(3)
C44-C43-C42	121.5(3)
C45-C44-C43	120.2(3)
C44-C45-C46	120.1(3)
C45-C46-C47	119.9(3)
C42-C47-C46	121.0(3)

Supplementary Table 6. Torsion angles [°] for **9**

C38-W1-N2-C7	119.2(8)
N1-W1-N2-C7	14.3(8)
N3-W1-N2-C7	-147.8(8)
N4-W1-N2-C7	-77.3(8)
C5-N1-C2-C3	-2.2(3)
W1-N1-C2-C3	169.40(18)
C5-N1-C2-C1	176.5(2)
W1-N1-C2-C1	-11.9(4)
N1-C2-C3-C4	1.4(3)
C1-C2-C3-C4	-177.2(3)
C2-C3-C4-C5	0.0(3)
C3-C4-C5-N1	-1.4(3)
C3-C4-C5-C6	170.5(2)
C2-N1-C5-C4	2.2(3)
W1-N1-C5-C4	-169.63(18)
C2-N1-C5-C6	-169.5(2)
W1-N1-C5-C6	18.6(4)
W1-N2-C7-C8	-62.3(9)
W1-N2-C7-C12	112.5(7)
N2-C7-C8-C9	172.4(2)
C12-C7-C8-C9	-2.3(4)
N2-C7-C8-C13	-6.9(4)
C12-C7-C8-C13	178.5(2)
C7-C8-C9-C10	-1.4(4)
C13-C8-C9-C10	177.9(3)
C8-C9-C10-C11	3.4(4)
C9-C10-C11-C12	-1.7(4)
C10-C11-C12-C7	-1.9(4)
C10-C11-C12-C16	176.7(3)
N2-C7-C12-C11	-170.9(2)
C8-C7-C12-C11	3.8(4)
N2-C7-C12-C16	10.6(4)
C8-C7-C12-C16	-174.7(2)
C9-C8-C13-C15	-64.3(3)
C7-C8-C13-C15	114.9(3)
C9-C8-C13-C14	57.8(3)

C7-C8-C13-C14	-123.0(3)
C11-C12-C16-C18'	-71.2(5)
C7-C12-C16-C18'	107.3(5)
C11-C12-C16-C17	19.5(5)
C7-C12-C16-C17	-162.0(3)
C11-C12-C16-C18	-101.8(3)
C7-C12-C16-C18	76.7(3)
C11-C12-C16-C17'	53.0(5)
C7-C12-C16-C17'	-128.5(4)
C23-N4-C19-C20	2.7(4)
W1-N4-C19-C20	174.0(2)
N4-C19-C20-C21	-0.4(4)
C19-C20-C21-C22	-1.3(4)
C20-C21-C22-C23	0.8(5)
C19-N4-C23-C22	-3.2(4)
W1-N4-C23-C22	-175.2(2)
C19-N4-C23-C24	172.2(2)
W1-N4-C23-C24	0.2(3)
C21-C22-C23-N4	1.5(4)
C21-C22-C23-C24	-173.6(3)
C28-N3-C24-C25	5.6(4)
W1-N3-C24-C25	-166.8(2)
C28-N3-C24-C23	-171.3(2)
W1-N3-C24-C23	16.3(3)
N4-C23-C24-N3	-10.3(4)
C22-C23-C24-N3	165.0(3)
N4-C23-C24-C25	172.9(3)
C22-C23-C24-C25	-11.9(4)
N3-C24-C25-C26	-3.4(4)
C23-C24-C25-C26	173.2(3)
C24-C25-C26-C27	-0.9(4)
C25-C26-C27-C28	3.0(4)
C24-N3-C28-C27	-3.5(4)
W1-N3-C28-C27	168.7(2)
C26-C27-C28-N3	-0.8(4)
O1-C29-C30-F2	-56.1(3)
C32-C29-C30-F2	-179.8(4)
C32'-C29-C30-F2	-177.1(6)

1143	C31-C29-C30-F2	60.9(3)
1144	O1-C29-C30-F1	63.2(3)
1145	C32-C29-C30-F1	-60.6(4)
1146	C32'-C29-C30-F1	-57.8(6)
1147	C31-C29-C30-F1	-179.8(2)
1148	O1-C29-C30-F3	-177.8(2)
1149	C32-C29-C30-F3	58.4(4)
1150	C32'-C29-C30-F3	61.2(6)
1151	C31-C29-C30-F3	-60.8(3)
1152	O1-C29-C31-F6	48.1(3)
1153	C32-C29-C31-F6	173.7(4)
1154	C32'-C29-C31-F6	171.2(6)
1155	C30-C29-C31-F6	-69.7(3)
1156	O1-C29-C31-F5	-70.7(3)
1157	C32-C29-C31-F5	54.8(5)
1158	C32'-C29-C31-F5	52.3(6)
1159	C30-C29-C31-F5	171.4(2)
1160	O1-C29-C31-F4	169.1(2)
1161	C32-C29-C31-F4	-65.3(5)
1162	C32'-C29-C31-F4	-67.8(6)
1163	C30-C29-C31-F4	51.3(3)
1164	O1-C29-C32-C37	-13.6(12)
1165	C31-C29-C32-C37	-135.5(12)
1166	C30-C29-C32-C37	107.1(12)
1167	O1-C29-C32-C33	157.8(4)
1168	C31-C29-C32-C33	35.9(5)
1169	C30-C29-C32-C33	-81.6(4)
1170	C37-C32-C33-C34	-7.3(14)
1171	C29-C32-C33-C34	-178.6(5)
1172	C32-C33-C34-C35	3.9(9)
1173	C33-C34-C35-C36	0.6(10)
1174	C34-C35-C36-C37	-1.6(14)
1175	C33-C32-C37-C36	6(2)
1176	C29-C32-C37-C36	178.3(13)
1177	C35-C36-C37-C32	-2(2)
1178	O1-C29-C32'-C37'	-19(2)
1179	C31-C29-C32'-C37'	-140.5(19)
1180	C30-C29-C32'-C37'	100(2)

O1-C29-C32'-C33'	179.1(7)
C31-C29-C32'-C33'	58.0(9)
C30-C29-C32'-C33'	-61.2(8)
C37'-C32'-C33'-C34'	12(2)
C29-C32'-C33'-C34'	174.0(8)
C32'-C33'-C34'-C35'	-9.2(15)
C33'-C34'-C35'-C36'	1.2(15)
C34'-C35'-C36'-C37'	3(2)
C35'-C36'-C37'-C32'	0(4)
C33'-C32'-C37'-C36'	-8(4)
C29-C32'-C37'-C36'	-170(2)
W1-C38-C39-C41	4.3(4)
W1-C38-C39-C42	128.4(3)
W1-C38-C39-C40	-115.0(3)
C38-C39-C42-C47	-119.1(3)
C41-C39-C42-C47	5.4(3)
C40-C39-C42-C47	123.9(3)
C38-C39-C42-C43	61.0(3)
C41-C39-C42-C43	-174.5(2)
C40-C39-C42-C43	-56.0(3)
C47-C42-C43-C44	-0.1(4)
C39-C42-C43-C44	179.8(3)
C42-C43-C44-C45	0.3(5)
C43-C44-C45-C46	-0.4(5)
C44-C45-C46-C47	0.2(4)
C43-C42-C47-C46	-0.1(4)
C39-C42-C47-C46	-180.0(2)
C45-C46-C47-C42	0.0(4)

----

2. Temperature dependence of the association constant for 9; estimation of the thermodynamic parameters of the association by van't Hoff analysis

Variable temperature NMR measurements were performed in order to obtain the thermodynamic parameters for the association of 2,2'-bipyridine and **5**, resulting in the formation of **9**. **9** was dissolved in benzene-*d6* in 0.01 and 0.05 M concentration and transferred to a sealed Young NMR tube. ^{1}H and ^{19}F NMR spectra were collected at various temperatures. The samples were allowed 30 minutes for equilibration at each temperature step. The temperature of the NMR probe was controlled with ± 0.1 K accuracy. At least 5T_1 delays were used between scans. Equilibrium constants of complexation (*K*) were calculated at every temperature using the average of the integrals of the alkylidene peaks in the ^{1}H spectra and the CF_3 peaks in the ^{19}F spectra according to the following expression:

$$K = \frac{I_{\text{complex}} \cdot c}{(I_{\text{free}} \cdot c)^2}$$

where *c* is the molar concentration of the complex in mol/dm^3. Thermodynamic parameters Δ*H* and Δ*S* were calculated from the fitted linear to RlnK against 1000/T (van't Hoff plot):

$$\text{Rln}(K) = \frac{-\Delta H}{T} + \Delta S$$

Gibb's free energy of complexation was calculated as follows:

$$\Delta G = \Delta H - T\Delta S$$

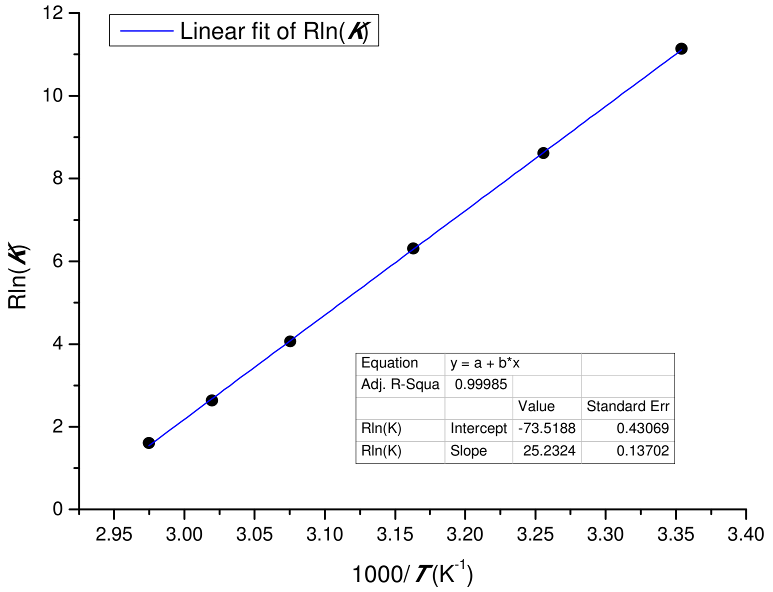

Supplementary Figure 13. van't Hoff plot derived from variable temperature ^{1}H and ^{19}F NMR spectra of the equilibrium between **5** and **9** in benzene-*d6* in the range of 298 K to 336 K (0.05M solution)

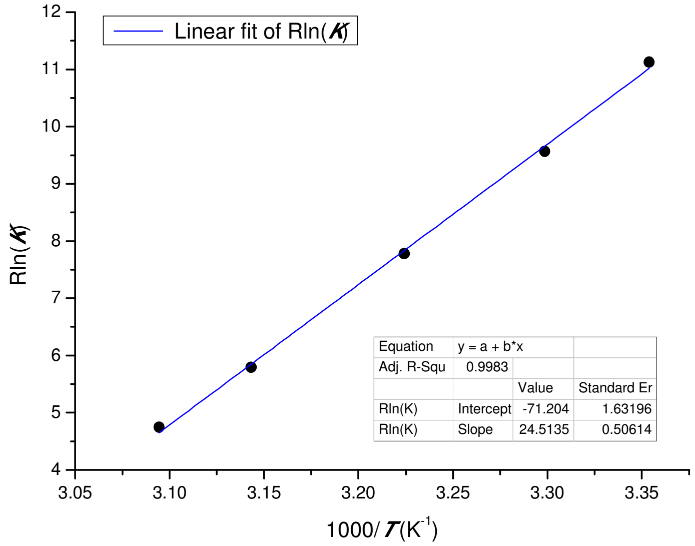

Supplementary Figure 14. van't Hoff plot derived from variable temperature ^{1}H and ^{19}F NMR spectra of the equilibrium between **5** and **9** in benzene-*d6* in the range of 298 K to 323 K (0.01 M solution)

Supplementary Table 7. Thermodynamic parameters of the complexation from van't Hoff analysis of variable-temperature NMR data

18-electron adduct	c (mol·dm^{-3})	ΔG_{298} (kcal·mol^{-1})	ΔH (kcal·mol^{-1})	ΔS (cal·mol^{-1}·K^{-1})
**9**	0.01	-3.3	-24.5	-71.2
**9**	0.05	-3.3	-25.2	-73.5

3. Computational details

3.1. General

The Gibbs free energies of the presented structures were calculated using density functional theory (DFT) with the dispersion-corrected, range-separated hybrid *ω*B97XD exchange-correlation functional.[3-5] The geometry optimizations were carried out at the *ω*B97XD/Def2SVP level, but for each optimized geometry, an additional single-point energy calculation was performed at the *ω*B97XD/Def2TZVPP level.[6] We note that this combination of functional and basis sets was shown to provide reasonable structural data and energetics for analogous Schrock-type complexes.[7] The thermal and entropic contributions were estimated within the RRHO (rigid rotor - harmonic oscillator) approximation for T = 298.15 K and c = 1 mol/dm^3 conditions. The solvent effects were taken into account as well by computing the solvation free energies (at the *ω*B97XD/Def2SVP level, using benzene as a solvent) via the SMD solvation model.[8] The relative stabilities reported in the manuscript refer to solvent phase Gibbs free energies as obtained form

$$G = E_0' + (G_0 - E_0) + (G_{sol} - E_0) + \Delta G_{conc}$$

where E_0 and E_0' refer to electronic energies computed at *ω*B97XD/Def2SVP and *ω*B97XD/Def2TZVPP level of DFT; G_0 and G_{sol} denote gas-phase Gibbs free energies and solvation free energies computed at *ω*B97XD/Def2SVP level, and ΔG_{conc} = 0.003019 hartree is the correction corresponding to the c = 1 mol/dm^3 concentration. The Gibbs free energies of complexation were corrected for the basis set superposition error (BSSE) as well, which were estimated via the counterpoise correction scheme[9] applied at *ω*B97XD/Def2TZVPP level. The computed BSSE corrections are 1.46 and 1.45 kcal/mol for complexes **7** and **9**, respectively, and they reduce the exergonicity of complexation. All DFT calculations were carried out with the *Gaussian16* software package.[10]

For the MAP complexes, we carried out an extensive conformational analysis via an initial Monte-Carlo (MC) search using an empirical force field (OPLS_2005), which was then followed by the standard DFT calculations described above. The MC conformational search was carried out using the *MacroModel* software.[11]

3.2. Conformers of 2,2'-bipyridine

The most stable form of the uncoordinated 2,2'-bipyridine (**bipy**) is planar and it has a *trans* arrangement of N atoms (Supplementary Figure 15, Conformational equilibrium of **bipy**). The *cis* isomer that acts as a bidentate ligand in the coordination to the metal center of MAP complexes is predicted to be 4.5 kcal/mol less stable. This conformer is distorted from the coplanar structure, which stems from the steric repulsion between the H atoms and also from the Coulomb interaction between the lone pairs of the N atoms. On the other hand, the coplanar *trans* structure is stabilized via intramolecular N···H interactions. The potential energy curve computed with respect to the N-C-C-N dihedral angle is shown in Supplementary Figure 15, the optimized structures are depicted in Supplementary Figure 16. It is apparent that the planar *cis* structure is a saddle-point on the potential energy curve and it is predicted to be 6.6 kcal/mol higher in free energy relative to the *trans* conformer.

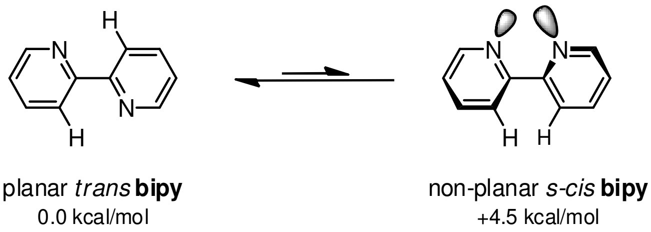

Conformational equilibrium of **bipy**.

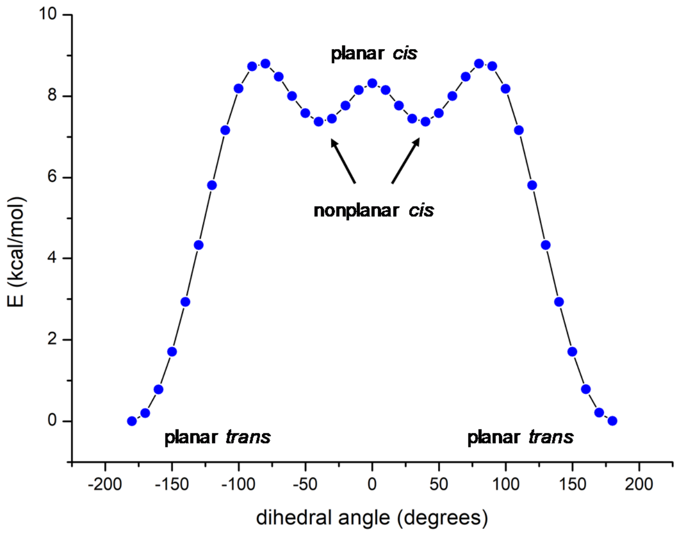

Supplementary Figure 15: Potential energy curve computed at the ωB97XD/Def2SVP level with respect to the N-C-C-N dihedral angle of **bipy**.

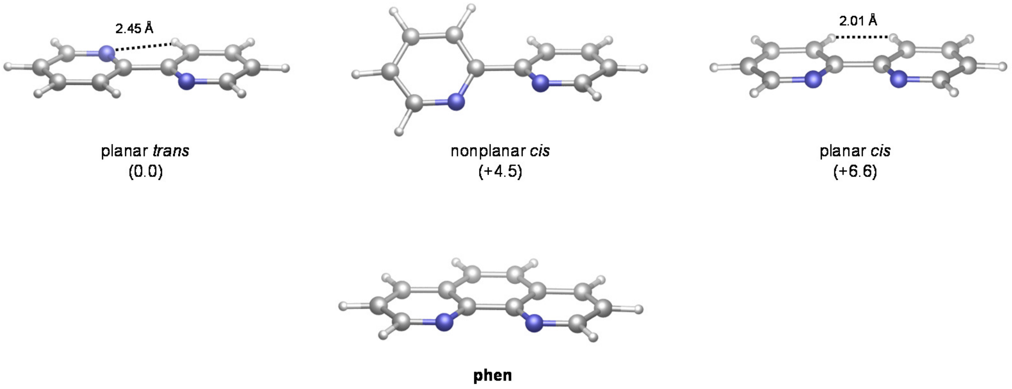

Supplementary Figure 16: Optimized structures of **bipy** conformers as obtained from ωB97XD/Def2SVP calculations. Relative stabilities are given in parenthesis (in kcal/mol). The structure of (1,10-phenanthroline) **phen** is also given for comparison.

3.3. Coordination of 1,10-phenatroline and 2,2'-bipyridine to complex 5

The Gibbs free energies of the coordination of ligands 1,10-phenanthroline (**phen**) and 2,2'-bipyridine (**bipy**) to complex **5** were computed using the methodology described in the previous section. The optimized structure of **5** and the adducts formed with **phen** and **bipy** are depicted in Supplementary Figure 17.

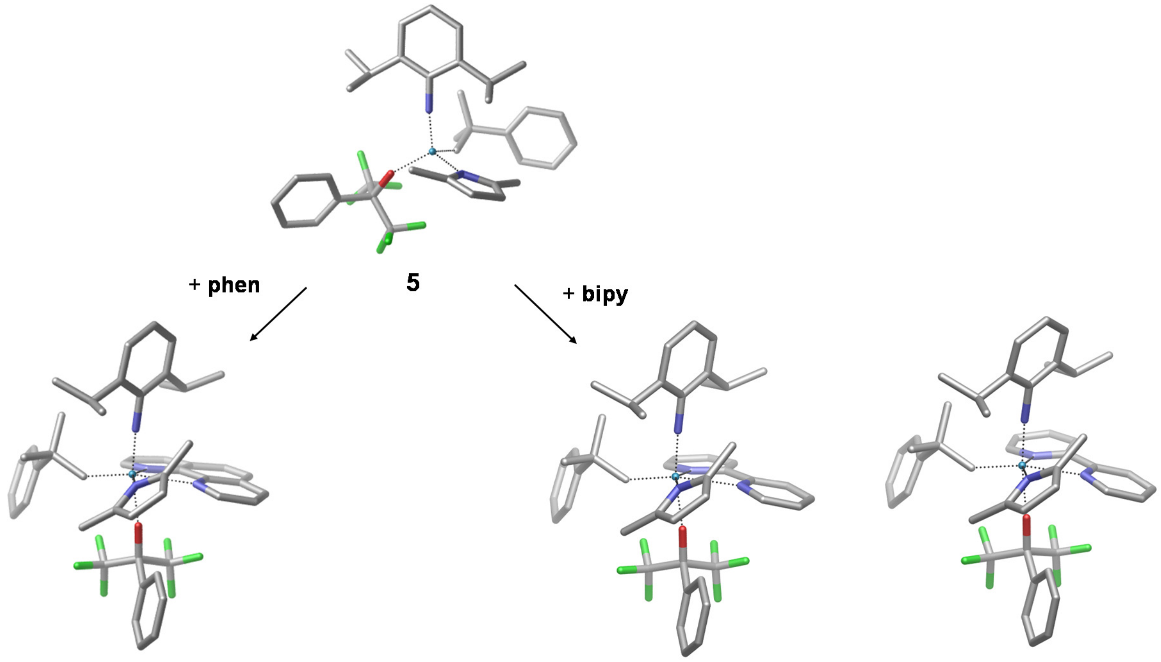

7 (-8.5) **9** (-1.8) **9** (Xray)

Supplementary Figure 17. Equilibrium geometries of complexes **5**, **7** and **9**. Free energies of association of the ligands **phen** and **bipy** to complex **5** are shown in parenthesis (in kcal/mol). The X-ray structure of **9** is also shown for comparison. All H atoms are omitted for clarity.

Computations predict hexacoordinated, quasi square bipyramidal structures for the adduct species **7** and **9**, wherein the arylimido and alkoxide ligands are aligned with the molecular axis, and the remaining three ligands (pyrrolide, alkylidine and the chelating **phen**/**bipy**) are in the equatorial position. The relative position and the orientation of the coordinated ligands are analogous in the most stable forms of the two adducts (**7** and **9**), and they are in full agreement with the X-ray structure of **9** (see Supplementary Figure 8).

Computations reveal a significant difference in the stability of **phen** and **bipy** complexes. The coordination of **phen** is found to be highly exergonic (ΔG = -8.5 kcal/mol), whereas the adduct formation with **bipy** is predicted to be close to thermodynamic equilibrium (ΔG = -1.8 kcal/mol). This trend can be easily rationalized in terms of the conformational change taking place upon the

coordination **bipy**: this ligand coordinates via the planar *cis* structure, which is 6.6 kcal/mol less stable than its ground state.

3.4. Total energy data

Supplementary Table 8. Various energy contributions computed for optimized structures (in hartree).[a]

	E_0	G_0	G_{sol}	E_0'	G
**bipy** - *trans*	-494.8580	-494.7312	-494.8705	-495.3856	-495.2688
**bipy** - *cis* nonplanar	-494.8463	-494.7199	-494.8605	-495.3761	-495.2617
**bipy** - *cis* planar	-494.8447	-494.7176	-494.8593	-495.3738	-495.2583
**phen**	-570.9989	-570.8596	-571.0151	-571.6043	-571.4784
complex **5**	-2284.0310	-2283.3797	-2284.0601	-2286.4913	-2285.8780
complex **7**	-2855.0977	-2854.2740	-2855.1359	-2858.1508	-2857.3722
complex **9**	-2778.9420	-2778.1316	-2778.9790	-2781.9185	-2781.1520

[a] For the definition of various energy terms, see the Computational details section.

Supplementary Table 9. Total energy data (in hartree) for BSSE calculations.[a]

	E_0'
complex **7**	-2858.1508
fragment 1 - full basis	-2286.4327
fragment 2 - full basis	-571.6038
fragment 1 - fragment basis	-2286.4314
fragment 2 - fragment basis	-571.6027
complex **9**	-2781.9185
fragment 1 - full basis	-2286.4318
fragment 2 - full basis	-495.3733
fragment 1 - fragment basis	-2286.4305
fragment 2 - fragment basis	-495.3722

[a]The counterpoise method has been applied at the ωB97XD/Def2TZVPP level. Fragment 1 is defined by the atoms of complex **5** (in the adduct species); fragment 2 is defined by the atoms of **phen** and **bipy** (in complexes **7** and **9**, respectively)

3.5. Cartesian coordinates

Cartesian coordinates of the optimized geometries are given below in standard XYZ format (units are in Å). The first lines indicate the total number of atoms, the second one is the labeling followed by the ωB97XD/Def2TZVPP electronic energy.

20

bipy *trans* - scf done: -495.385588

C	-1.171308	2.707297	-0.000044
C	-0.011180	3.481167	-0.000033
C	1.216546	2.822549	-0.000099
C	1.234448	1.432632	-0.000118
C	0.013933	0.746760	-0.000126
N	-1.162749	1.378946	-0.000069
C	-1.234448	-1.432632	-0.000118
C	-0.013933	-0.746760	-0.000126
C	-1.216546	-2.822549	-0.000099
C	0.011180	-3.481167	-0.000033
C	1.171308	-2.707297	-0.000044
N	1.162749	-1.378946	-0.000069
H	-2.155619	3.188180	0.000015
H	-0.073787	4.570693	0.000018
H	2.151752	3.386991	-0.000107
H	-2.151752	-3.386991	-0.000107
H	0.073787	-4.570693	0.000018
H	2.155619	-3.188180	0.000015
H	2.160965	0.858966	-0.000137
H	-2.160965	-0.858966	-0.000137

20

bipy *cis* nonplanar - scf done: -495.376118

C	0.002400	0.748000	0.003700
C	-0.371200	2.857000	1.098600
C	0.037500	3.483900	-0.074400
C	0.406200	2.679300	-1.153600
C	-0.002400	-0.748000	0.003700
C	0.371200	-2.857000	1.098600
C	-0.037500	-3.483900	-0.074400
C	-0.406200	-2.679300	-1.153600
H	-0.680200	3.441700	1.967900
H	0.068200	4.571600	-0.158100
H	0.733500	3.137300	-2.093700
H	0.680200	-3.441700	1.967900
H	-0.068200	-4.571600	-0.158100
H	-0.733500	-3.137300	-2.093700
N	-0.389300	-1.352100	-1.121400
N	0.389300	1.352100	-1.121400
C	0.389300	-1.466200	1.141600
H	0.725400	-0.942300	2.038200
C	-0.389300	1.466200	1.141600
H	-0.725400	0.942300	2.038200

20

bipy *cis* planar - scf done: -495.373784

C	0.751400	0.000000	0.049700
C	2.882200	0.000000	1.176700
C	3.497400	0.000000	-0.070700
C	2.675800	0.000000	-1.198400
C	-0.751400	0.000000	0.049700
C	-2.882200	0.000000	1.176700

C -3.497400 0.000000 -0.070700
C -2.675800 0.000000 -1.198400
H 3.475100 0.000000 2.094000
H 4.583900 0.000000 -0.172600
H 3.118400 0.000000 -2.201100
H -3.475100 0.000000 2.094000
H -4.583900 0.000000 -0.172600
H -3.118400 0.000000 -2.201100
N -1.351000 0.000000 -1.142600
N 1.351000 0.000000 -1.142600
C -1.492700 0.000000 1.241100
H -1.002900 0.000000 2.214400
C 1.492700 0.000000 1.241100
H 1.002900 0.000000 2.214400

22
phen - scf done: -571.604337
C -2.692600 -1.549400 0.000000
C -3.474600 -0.376400 0.000000
C -2.826400 0.838100 0.000000
C -1.415200 0.868700 0.000000
C -0.730600 -0.373000 0.000000
N -1.378200 -1.552300 0.000000
C -0.678100 2.103600 0.000000
C 0.678100 2.103600 0.000000
C 1.415200 0.868700 0.000000
C 0.730600 -0.373000 0.000000
C 2.826400 0.838100 0.000000
C 3.474600 -0.376400 0.000000
C 2.692600 -1.549400 0.000000
N 1.378200 -1.552300 0.000000
H -3.184000 -2.529600 0.000000
H -4.564100 -0.441200 0.000000
H -3.385700 1.777400 0.000000
H -1.234800 3.044000 0.000000
H 1.234800 3.044000 0.000000
H 3.385700 1.777400 0.000000
H 4.564100 -0.441200 0.000000
H 3.184000 -2.529600 0.000000

89
complex **5** - scf done: -2286.491273
C 0.999672 -1.186807 -0.927863
W 0.094079 -0.124357 0.324884
N 0.690663 -0.770018 2.147594
C 1.629027 -1.684965 2.625331
C 1.610507 -1.653900 3.995764
C 0.636306 -0.689465 4.399053
C 0.092655 -0.158517 3.261961
C 2.481886 -2.517558 1.730809
C -0.968966 0.890133 3.127510
N 0.786602 1.434366 0.021927
C 1.453682 2.626968 -0.187191
C 0.810840 3.656389 -0.909101
C 1.514955 4.841598 -1.126189
C 2.815043 4.997884 -0.654876
C 3.440228 3.964983 0.038805
C 2.781802 2.760044 0.285889
C -0.621033 3.470665 -1.376813
C 3.435198 1.627600 1.058466
O -1.828823 -0.205187 0.063662
C -2.940851 -0.887958 -0.358838
C -2.829095 -1.090516 -1.899592
F -2.568470 0.076477 -2.478854
C -2.937113 -2.255428 0.388035
F -3.248908 -2.080124 1.664800
F -1.838552 -1.931110 -2.209940

F	-3.943026	-1.579429	-2.435754
F	-3.787097	-3.133521	-0.139404
F	-1.716404	-2.809524	0.353960
H	0.526304	-2.182976	-0.761892
H	3.048000	0.685603	0.637127
H	4.463973	4.101889	0.391236
H	3.350086	5.932671	-0.835688
H	1.044077	5.657533	-1.677233
H	0.361989	-0.415774	5.416344
H	2.234392	-2.265397	4.645296
H	-1.250003	1.253090	4.124449
H	-0.622297	1.768186	2.552552
H	-1.886326	0.509595	2.647938
H	1.884262	-3.165293	1.071507
H	3.123669	-1.904895	1.080225
H	3.129928	-3.163459	2.337629
C	1.956752	-1.159246	-2.107301
C	-4.212065	-0.099245	-0.025591
C	-4.077251	1.198363	0.467368
C	-5.492152	-0.636088	-0.202155
C	-5.204375	1.956491	0.776806
H	-3.079914	1.610581	0.606101
C	-6.616746	0.122235	0.112521
H	-5.620999	-1.648002	-0.584852
C	-6.477661	1.420722	0.600470
H	-5.081639	2.971820	1.159818
H	-7.610447	-0.308190	-0.026632
H	-7.362371	2.012538	0.844547
C	1.270137	-1.823585	-3.317809
H	0.906950	-2.836194	-3.094287
H	0.399231	-1.226915	-3.624661
H	1.969753	-1.888729	-4.165045
C	2.301656	0.280004	-2.503630
H	2.759538	0.847296	-1.685853
H	2.994369	0.285662	-3.358650
H	1.387490	0.817693	-2.793716
C	3.200750	-1.953956	-1.675490
C	3.143746	-3.349262	-1.554301
C	4.396070	-1.319467	-1.319055
C	4.230819	-4.080300	-1.083421
H	2.226999	-3.882836	-1.813508
C	5.488002	-2.047142	-0.845956
H	4.487146	-0.236903	-1.397122
C	5.410617	-3.431421	-0.721612
H	4.152973	-5.166165	-0.993896
H	6.404991	-1.520751	-0.571178
H	6.263265	-4.002308	-0.348072
H	-0.751677	2.400452	-1.602376
C	-0.963780	4.247388	-2.647399
H	-1.962550	3.957185	-3.006396
H	-0.984990	5.335038	-2.474382
H	-0.240511	4.044381	-3.451281
C	-1.589266	3.819515	-0.239161
H	-2.632004	3.641235	-0.543556
H	-1.381022	3.214391	0.656535
H	-1.489163	4.879519	0.043058
C	3.015019	1.670012	2.533099
H	3.352390	0.766920	3.064262
H	3.443213	2.554622	3.030952
H	1.923086	1.718037	2.638172
C	4.956730	1.583083	0.922981
H	5.341403	0.649496	1.360394
H	5.275003	1.622640	-0.130437
H	5.444220	2.416289	1.453572

111

complex **7** - scf done: -2858.150829

W	-9.851913	6.668967	3.692285
N	-9.836772	8.309361	4.307705
O	-9.968807	4.640324	3.339297
C	-8.667937	6.913786	2.210294
N	-11.780675	6.916215	2.851491
C	-10.060462	9.599969	4.752042
C	-9.841988	3.325882	3.062175
H	-8.632705	5.948372	1.684850
C	-7.648878	7.879907	1.615292
C	-12.222497	6.500408	1.607464
C	-12.907696	7.297171	3.579427
C	-10.577618	10.581750	3.867970
C	-9.827700	9.904625	6.116972
C	-9.609123	2.538577	4.391369
C	-8.585193	3.156856	2.149204
C	-11.105252	2.741461	2.402700
C	-8.182211	8.369314	0.250767
C	-7.414362	9.095421	2.513059
C	-6.336133	7.098215	1.383279
C	-11.316575	6.030020	0.518904
C	-13.598831	6.578601	1.556423
C	-14.034491	7.072399	2.811760
C	-12.904137	8.060349	4.875456
C	-10.864413	11.845434	4.393288
C	-10.837547	10.323648	2.388217
C	-10.129654	11.181218	6.584797
C	-9.215778	8.850560	7.019162
F	-10.636262	2.729087	5.221755
F	-8.504960	2.947238	5.030329
F	-9.488729	1.224776	4.207475
F	-8.190705	1.889392	1.988334
F	-8.828205	3.653410	0.932888
F	-7.541419	3.820749	2.647411
C	-11.122101	1.528324	1.704681
C	-12.297508	3.448081	2.562012
H	-7.452785	9.041585	-0.227340
H	-9.126004	8.914905	0.387797
H	-8.374082	7.531991	-0.434894
H	-6.693080	9.791854	2.059392
H	-7.047654	8.809117	3.509310
H	-8.350603	9.633963	2.669407
C	-6.331773	5.952854	0.572505
C	-5.121066	7.473179	1.970721
H	-11.911219	5.805456	-0.376821
H	-10.768461	5.118827	0.795907
H	-10.572332	6.797341	0.257296
H	-14.213499	6.305587	0.700132
H	-15.054929	7.307978	3.112726
H	-13.865228	7.908979	5.389134
H	-12.793969	9.142700	4.703357
H	-12.103217	7.785693	5.571065
H	-11.274818	12.612296	3.731943
C	-10.651891	12.149777	5.731798
H	-10.455594	9.320634	2.152981
C	-10.106104	11.344894	1.505110
C	-12.330575	10.319516	2.041768
H	-9.959631	11.426867	7.634802
H	-9.593552	7.884765	6.660349
C	-9.611918	8.961012	8.490056
C	-7.692021	8.845154	6.851055
H	-10.209338	0.947567	1.578127
C	-12.310993	1.049756	1.159071
C	-13.485510	2.968967	2.016169
H	-12.288172	4.398756	3.091307
H	-7.256775	5.616566	0.100541
C	-5.175772	5.206985	0.370654
C	-3.956522	6.727106	1.772898

H	-5.066162	8.360523	2.601646
H	-10.891659	13.144763	6.112801
H	-10.209196	11.071824	0.443421
H	-9.033326	11.406992	1.737319
H	-10.531228	12.354137	1.622084
H	-12.463235	10.150247	0.962167
H	-12.802574	11.283538	2.292855
H	-12.870758	9.517277	2.557987
H	-9.176612	9.849844	8.973701
H	-9.246860	8.080492	9.042331
H	-10.704807	9.009399	8.609269
H	-7.417328	8.713476	5.794719
H	-7.229192	8.032845	7.433992
H	-7.262588	9.799546	7.194608
H	-12.307667	0.106532	0.608371
C	-13.496400	1.768020	1.311595
H	-14.397670	3.557083	2.135140
H	-5.214038	4.315330	-0.258998
C	-3.977563	5.587990	0.974856
H	-3.025357	7.048772	2.245939
H	-14.425584	1.391755	0.877569
H	-3.068965	5.001978	0.820669
H	-12.763919	5.516678	5.173619
C	-12.055569	5.290799	5.976067
N	-10.797162	5.624032	5.760061
C	-12.486584	4.686021	7.168801
C	-9.867249	5.307062	6.683490
C	-11.562525	4.428383	8.154521
H	-13.540745	4.438647	7.294580
C	-8.476268	5.514137	6.347556
C	-10.202687	4.724175	7.922252
H	-11.861127	3.975328	9.102709
C	-7.483315	5.151876	7.280433
N	-8.168121	5.984770	5.113466
C	-9.168548	4.423184	8.872506
C	-6.134202	5.281607	6.891781
C	-7.864392	4.633706	8.565595
C	-6.892289	6.064806	4.763578
H	-9.453235	4.001648	9.838742
C	-5.839372	5.725692	5.624540
H	-5.342464	5.009166	7.593438
H	-7.076428	4.386449	9.279834
H	-6.680864	6.404310	3.751591
H	-4.814442	5.817564	5.265147

109

complex **9** - scf done: -2781.918451

C	-8.388368	3.412245	10.311640
C	-7.411219	3.606720	9.335372
C	-6.090064	3.237004	9.613219
C	-5.766267	2.661570	10.839823
C	-6.751274	2.457651	11.805416
C	-8.064181	2.836166	11.537047
C	-7.848674	4.276026	8.018596
C	-7.411487	5.773162	8.095656
F	-8.021166	6.375006	9.118095
O	-9.183327	4.213745	7.827499
W	-11.146610	4.089532	7.198347
N	-12.840582	4.279090	6.792244
C	-14.219409	4.350399	6.703031
C	-15.012860	3.191041	6.902499
C	-16.402916	3.342254	6.890061
C	-17.005829	4.576445	6.684864
C	-16.215486	5.702597	6.471961
C	-14.825702	5.611465	6.475450
C	-14.428515	1.802130	7.132221
C	-14.685432	1.281051	8.550424

1666	C	-13.949829	6.815976	6.189912
1667	C	-13.550793	6.821144	4.709704
1668	C	-10.699722	2.752728	5.904625
1669	C	-11.202974	2.073103	4.634661
1670	C	-11.414891	0.573334	4.937301
1671	C	-10.103801	2.209479	3.556688
1672	C	-10.328181	2.839185	2.325780
1673	C	-9.306071	2.980498	1.383949
1674	C	-8.032269	2.488098	1.648352
1675	C	-7.793847	1.845868	2.863454
1676	C	-8.815014	1.709495	3.797338
1677	C	-12.526266	2.664564	4.147621
1678	N	-10.493119	5.850978	5.848216
1679	C	-10.471557	7.115835	6.310046
1680	C	-10.207118	8.188156	5.457099
1681	C	-9.917442	7.951959	4.119291
1682	C	-9.891495	6.640722	3.665134
1683	C	-10.191032	5.626419	4.563634
1684	C	-10.649458	7.308168	7.775464
1685	C	-10.444881	8.543818	8.393098
1686	C	-10.495282	8.624867	9.779648
1687	C	-10.722977	7.467624	10.512721
1688	C	-10.931422	6.279811	9.821902
1689	N	-10.926526	6.211477	8.490853
1690	N	-11.609965	2.967641	8.934497
1691	C	-11.000603	1.809528	9.386468
1692	C	-11.387572	1.562180	10.687260
1693	C	-12.281269	2.597444	11.060177
1694	C	-12.424926	3.430479	9.967336
1695	C	-10.059834	0.990284	8.567473
1696	C	-13.471469	4.503986	9.850092
1697	C	-7.162473	3.611013	6.782481
1698	F	-5.841203	3.804286	6.717031
1699	F	-7.368647	2.290901	6.797146
1700	F	-7.672975	4.080984	5.643730
1701	C	-14.966926	0.792231	6.108272
1702	C	-14.554109	8.155032	6.607135
1703	F	-7.742075	6.449518	6.987248
1704	F	-6.100105	5.929360	8.270683
1705	H	-9.648996	2.486257	6.088941
1706	H	-11.744335	0.042361	4.030593
1707	H	-12.180829	0.449244	5.715095
1708	H	-10.491595	0.095993	5.294559
1709	H	-12.882300	2.149128	3.242807
1710	H	-12.443806	3.739006	3.929717
1711	H	-13.292377	2.560129	4.917696
1712	H	-9.753063	0.106555	9.143058
1713	H	-9.151772	1.545155	8.293733
1714	H	-10.532765	0.648530	7.634573
1715	H	-11.054702	0.721370	11.293754
1716	H	-12.821789	2.698962	12.000809
1717	H	-13.689295	4.909718	10.849158
1718	H	-14.414881	4.102083	9.447893
1719	H	-13.200911	5.342773	9.198369
1720	H	-17.031483	2.463783	7.054557
1721	H	-13.338622	1.875015	7.013233
1722	H	-16.694485	6.668906	6.303655
1723	H	-13.035562	6.678513	6.782877
1724	H	-5.302875	3.391642	8.876535
1725	H	-9.419149	3.685502	10.096301
1726	H	-8.590376	1.218521	4.745907
1727	H	-11.313624	3.237306	2.083370
1728	H	-18.094498	4.662533	6.687464
1729	H	-14.448499	-0.172928	6.218191
1730	H	-14.833122	1.133523	5.071893
1731	H	-16.040960	0.601419	6.259850
1732	H	-14.251684	0.275698	8.663770

H	-15.765156	1.208647	8.760517
H	-14.220049	1.916101	9.313189
H	-15.416800	8.435360	5.982326
H	-13.804550	8.954871	6.498536
H	-14.884467	8.138076	7.656590
H	-13.067308	5.873301	4.433599
H	-12.853293	7.643677	4.486225
H	-14.440310	6.943543	4.071746
H	-4.733024	2.369150	11.039487
H	-8.855884	2.672995	12.271035
H	-6.800624	1.452670	3.091219
H	-9.516216	3.476810	0.433238
H	-6.493868	2.000981	12.763817
H	-7.231209	2.599650	0.914479
H	-11.108334	5.340903	10.353354
H	-10.735754	7.470959	11.602650
H	-10.332143	9.581650	10.279099
H	-10.176712	4.587721	4.242880
H	-9.645667	6.383827	2.634770
H	-10.219564	9.434425	7.809857
H	-10.216073	9.208566	5.834323
H	-9.704080	8.785290	3.447399

4. Ring-closing-metathesis of diethyl diallylmalonate and homo-cross-metathesis of methyl oleate

4.1. General

The reactions were performed in a nitrogen-filled glovebox. Benzene used for the catalytic reactions was dried and stored as described in Chapter 1.1. prior to use. DCM (Merck, premium-grade) was used as received.

Analysis of metathesis reactions was performed by GC-MS-FID (Shimadzu GCMS QP2010Plus) or Agilent DB-23 (30 m × I.D.: 0.25 mm); the results of the reactions are given as the ratio of the areas of the corresponding GC-FID peaks.

4.2. Ring-closing-metathesis of diethyl diallylmalonate; catalytic reactions and analysis

Into 4-mL vials equipped with magnetic stirring bars, diethyl diallylmalonate (97 microL, 96 mg, 0.4 mmol; 242 microL, 240 mg, 1 mmol; 484 microL, 481 mg, 2 mmol; or 5 mmol), benzene, and 200 microL of 0.01 M benzene stock solutions of **1** or **11** (0.002 mmol) were transferred. The quantity of benzene was chosen to dilute a given reaction mixture to a total volume of 1 mL. (The volumes were assumed additive.) In the case of 5 mmol substrate, no benzene was added, except for the stock solution of the catalysts. The vials were closed with a cap with punctured septa to ensure pressure release, and the reaction mixtures were stirred at room temperature for 24 hours. Samples were taken after 1 hour, 4 hours, and 24 hours. They were quenched with undried analytical-grade dichloromethane, and analyzed by gas chromatography.

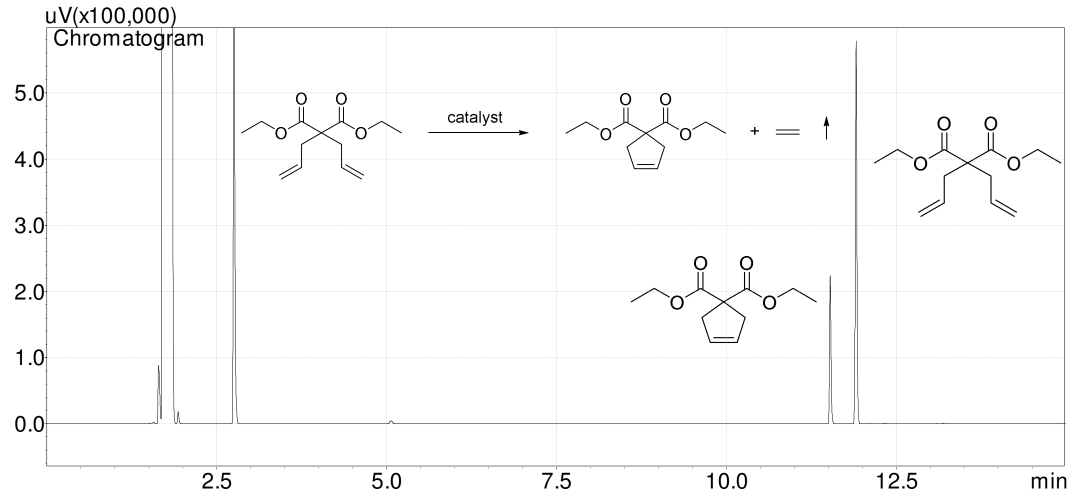

Supplementary Figure 18. Gas chromatogram of ring-closing-metathesis of diethyl diallylmalonate (2 mmol) with **11** (0.002 mmol) in 1 mL benzene solution; 1-hour reaction time at RT, 27% analytical yield

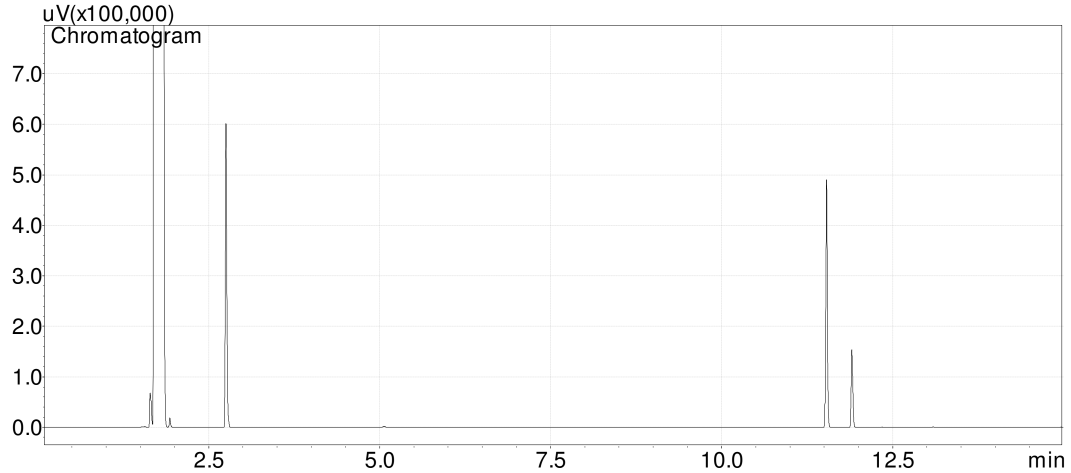

Supplementary Figure 19. Gas chromatogram of ring-closing-metathesis of diethyl diallylmalonate (2 mmol) with **11** (0.002 mmol) in 1 mL benzene solution; 4-hour reaction time at RT, 77% analytical yield

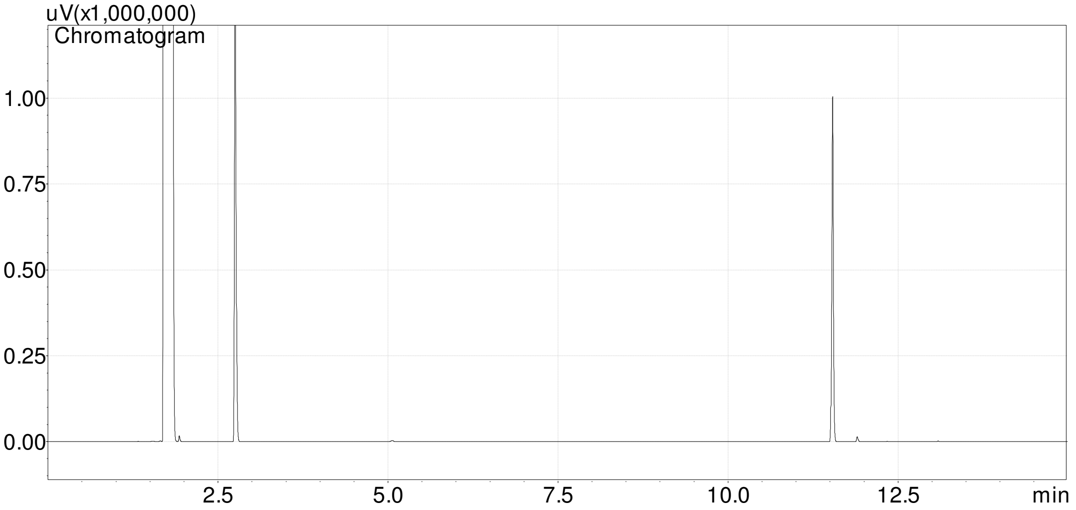

Supplementary Figure 20. Gas chromatogram of ring-closing-metathesis of diethyl diallylmalonate (2 mmol) with **11** (0.002 mmol) in 1 mL benzene solution; 24-hour reaction time at RT, 99% analytical yield

4.3. Homo-cross-metathesis of methyl oleate; catalytic reactions and analysis

In a glovebox, purified methyl oleate (339 microL, 296 mg, 1 mmol) was weighed into an oven-dried 4-mL vial by automatic pipette. A stock solution of the catalyst, **1** or **11**, (0.01 M in benzene, 50 microL, 0.0005 mmol) was added to the substrate. The vial was sealed, and the reaction mixture was stirred at 80°C for 4 hours. The reaction mixture was quenched with MeOH (100 microL). A sample was taken (2 microL), it was diluted with DCM (1 mL), and the solution was analyzed with GC.

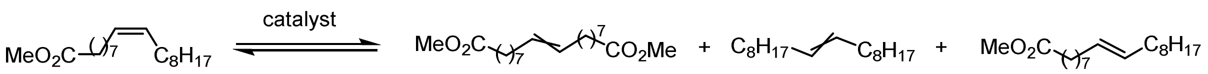

MO **9ODDAME** **OD** **ME**

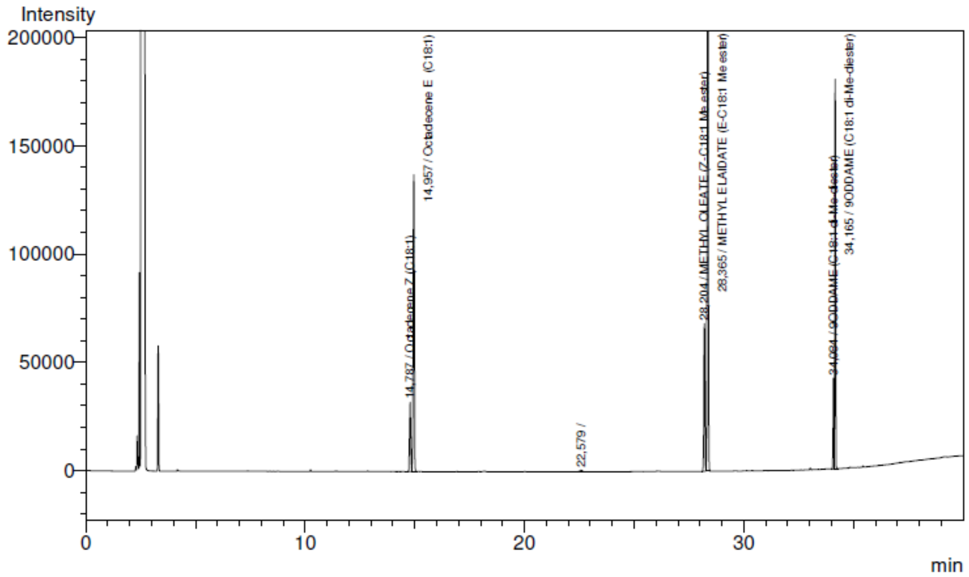

Peak no	etention tir	Area	Height	ID#	Name
1	14,787	121936	32018	2	Octadecene Z (C18:1)
2	14,957	520985	136722	3	Octadecene E (C18:1)
3	22,579	4943	744		
4	28,204	232328	68064	4	METHYL OLEATE (Z-C18:1 Me es
5	28,365	982271	291291	5	METHYL ELAIDATE (E-C18:1 Me (
6	34,084	107889	41767	6	9ODDAME (C18:1 di-Me-diester)
7	34,165	462862	178170	7	9ODDAME (C18:1 di-Me-diester)
Total		2433214	748776		

Supplementary Figure 21. Gas chromatogram of homo-cross-metathesis of methyl oleate (1 mmol) with **1** (0.0005 mmol) added to the substrate in 50 microL 0.01 M benzene solution; 4-hour reaction time at 80 °C, 90% conversion

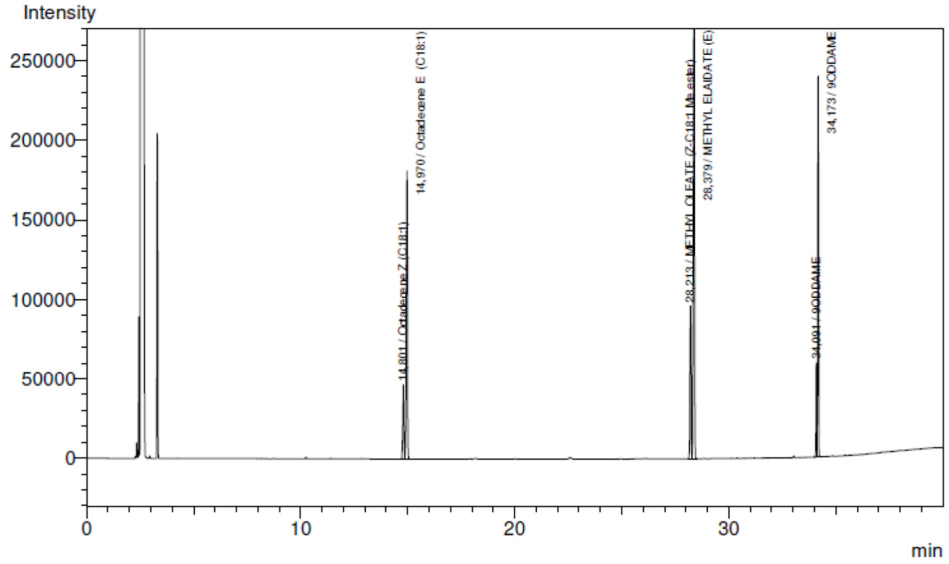

Peak no	etention tin	Area	Height	ID#	Name
1	14,801	172854	46624	2	Octadecene Z (C18:1)
2	14,970	699543	180850	3	Octadecene E (C18:1)
3	28,213	331706	96310	4	METHYL OLEATE (Z-C18:1 Me es
4	28,379	1332258	377338	5	METHYL ELAIDATE (E)
5	34,091	152178	59100	6	9ODDAME
6	34,173	636293	238368	7	9ODDAME
Total		3324832	998590		

Supplementary Figure 22. Gas chromatogram of homo-cross-metathesis of methyl oleate (1 mmol) with **11** (0.0005 mmol) added to the substrate in 50 microL 0.01 M benzene solution; 4-hour reaction time at 80 °C, 90% conversion

5. Ring-opening-metathesis-polymerization of norbornene

5.1. General

All the synthetic operations were carried out in a glove-box (nitrogen atmosphere, O_2<1 ppm, and H_2O<1 ppm) or under an argon atmosphere by using standard Schlenk techniques. Toluene was purified using a solvent purification system (Glass Conter or MBraun). *p*-Xylene (Wako Chemicals) and cyclooctane (Wako Chemicals) were degassed and stored over molecular sieves. Norbornene (Tokyo Kasei) was stored as a toluene solution over molecular sieves 3A. 1-Octene (Wako Chemicals) was distilled from calcium hydride. *p*-Tos-$NHNH_2$ (Aldrich) was used as received.

5.2. Analyses of Polymerization and Polymer

^1H and ^{13}C NMR spectra were recorded on a JEOL JNM-EX400WB spectrometer (399.78 MHz for ^1H, 100.53 MHz for ^{13}C) equipped with high-temperature probe CH8HT at 25–210 °C or on a Bruker Avance III 500MHz spectrometer (500.13 MHz for ^1H, 125.77 MHz for ^{13}C) with Cryoprobe™ DCH 500/3 at 25–60 °C. Chemical shifts were determined with reference to the tetramethylsilane (δ 0.00 ppm) or chloroform (δ 7.24 ppm for ^1H, δ 77.2 ppm for ^{13}C). *Cis*/*trans* ratios of the poly(NB)s were calculated by the integration ratios of each peak of ^1H NMR spectra, and meso/racemo ratios were estimated by peak separation of each peak of the ^{13}C NMR spectra of the hydrogenated poly(NB)s using Lorentzian function on JEOL Delta v5.0.2 NMR software. The molecular weight distributions (MWD) of the polymers were estimated on a gel-permeation chromatograph (GPC) (Tosoh HLC-8220 GPC; eluent tetrahydrofuran). The relative number- and weight-average molecular weights (M_n and M_w, respectively) were acquired by the use of a calibration curve obtained using polystyrene standards. Analysis of monomer conversion was performed by GC (Shimadzu GC-2010 Plus); the results of the reactions are given as the ratio of the areas of the corresponding GC peaks.

5.3. Polymerization

Polymerization was carried out in a pre-baked ampoule tube equipped with a rubber septum at 25 °C. A toluene solution of NB, 1-octene and cyclooctane was added to a toluene solution of catalyst at the prescribed temperature. In predetermined intervals, the aliquots were taken from the reaction mixture, and the reactions were terminated with small amounts of methanol. Monomer conversion was determined from residual monomer concentration measured by gas chromatography with cyclooctane as an internal standard. The obtained polymer was reprecipitated from methanol and dried *in vacuo* at 40 °C for 24 h.

5.4. Hydrogenation

A *p*-xylene solution of poly(NB) and a *p*-xylene solution of 4-folds of *p*-Tos-$NHNH_2$ were mixed in a glass flask equipped with a three-way stopcock. The mixture was heated to the reaction temperature while stirring. Chemical transfer hydrogenation was carried out at 125 °C for 5 h. After the hydrogenation reaction, the reaction mixture was cooled slowly to room temperature. The obtained polymer was reprecipitated from methanol and dried in vacuo at 40 °C for 24 h.

Supplementary Table 10. Polymerization of NB by **5** or **9** and Stereostructures of the Obtained Poly(NB)s [a]

Catalyst	Monomer conversion	M_n	M_w/M_n	cis/trans	meso/racemo [b]
**5**	>99%	13,500	2.05	89/11	14/86
**9**	>99%	203,000	4.67	96/4	0/100

[a] Polymerized in toluene at 25 °C; [NB] = 0.2 M, [NB]/[1-octene]/[**5** or **9**] = 200/40/1 mol/mol/mol. Monomer conversions were determined using GC; Cyclooctane was employed as an internal standard for NB. [b] Determined for the hydrogenated poly(NB)s.

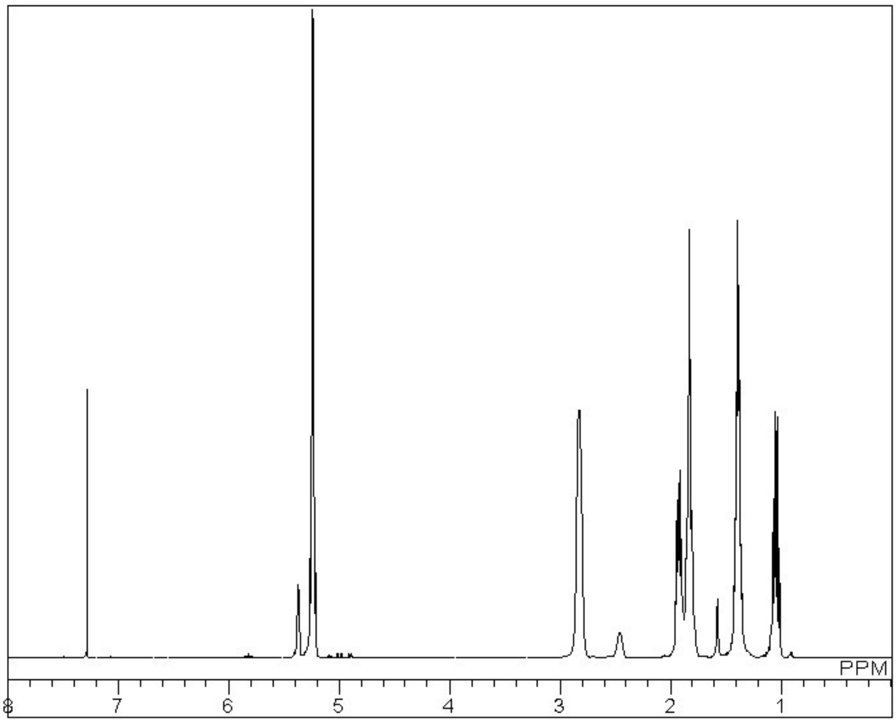

Supplementary Figure 23. ^{1}H NMR spectrum of the poly(NB) from **5** shown in **Supplementary Table 10** (recorded in $CDCl_3$ at 27 °C).

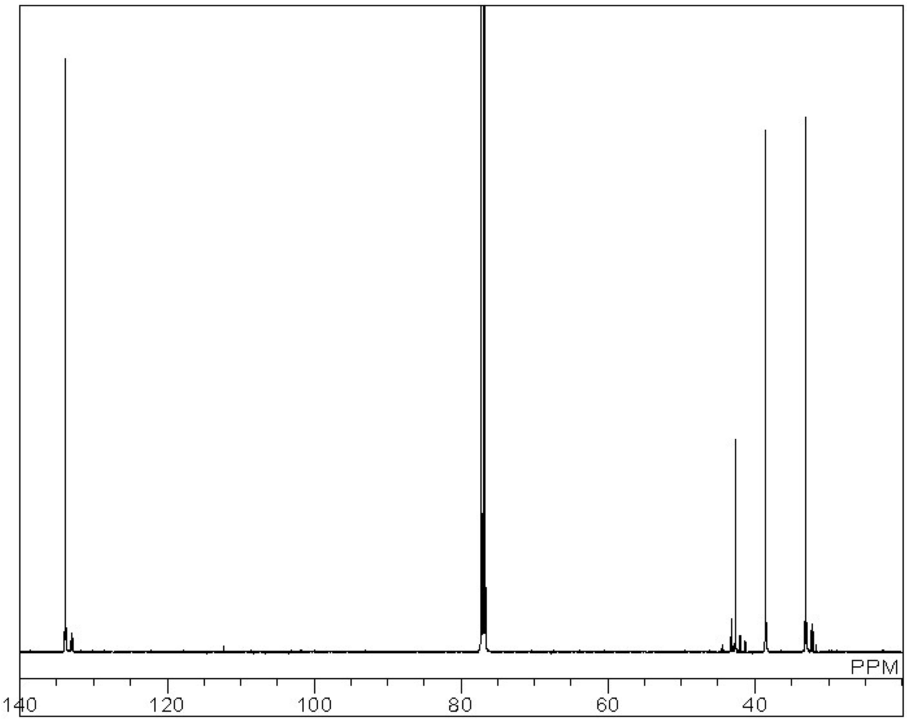

Supplementary Figure 24. ^{13}C NMR spectrum of the poly(NB) from **5** shown in **Supplementary Table 10** (recorded in $CDCl_3$ at 27 °C)

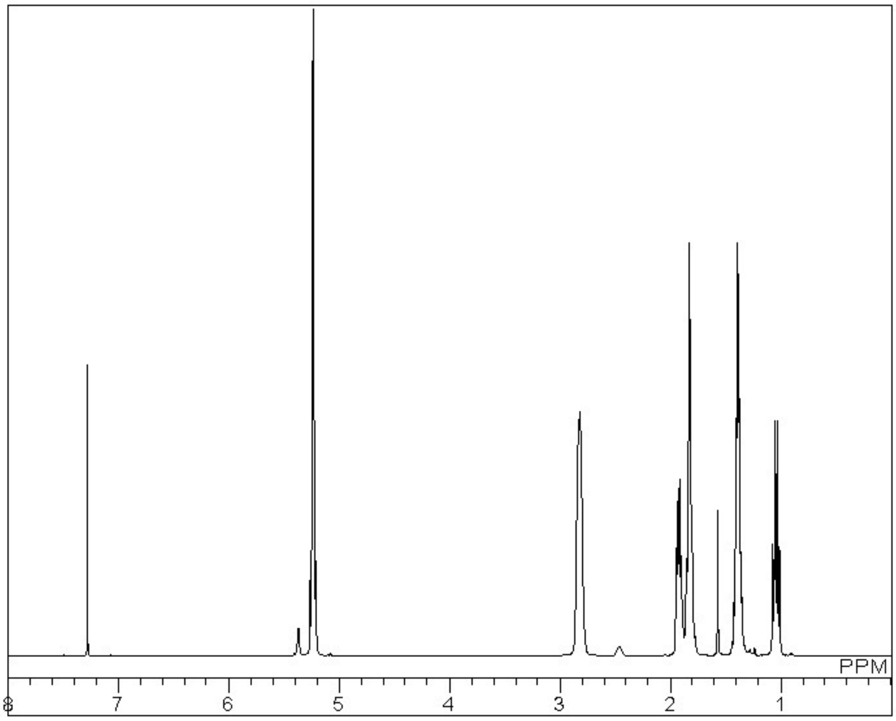

Supplementary Figure 25. ^{1}H NMR spectrum of the *cis*-, syndio-poly(NB) from **9** shown in **Supplementary Table 10** (recorded in $CDCl_3$ at 27 °C).

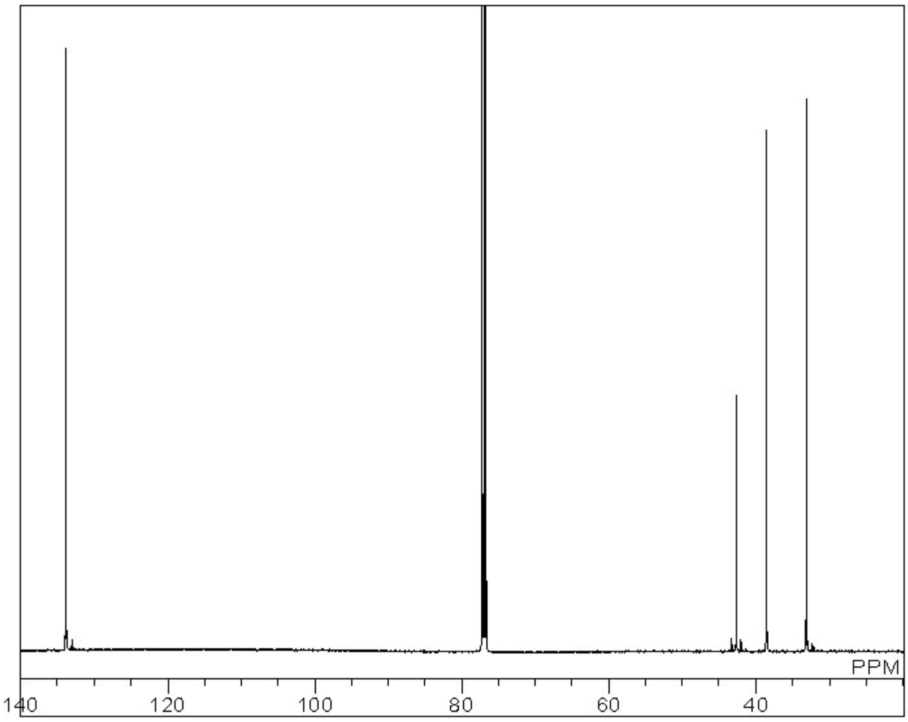

Supplementary Figure 26. ^{13}C NMR spectrum of the *cis*-, syndio-poly(NB) from **9** shown in **Supplementary Table 10** (recorded in $CDCl_3$ at 27 °C).

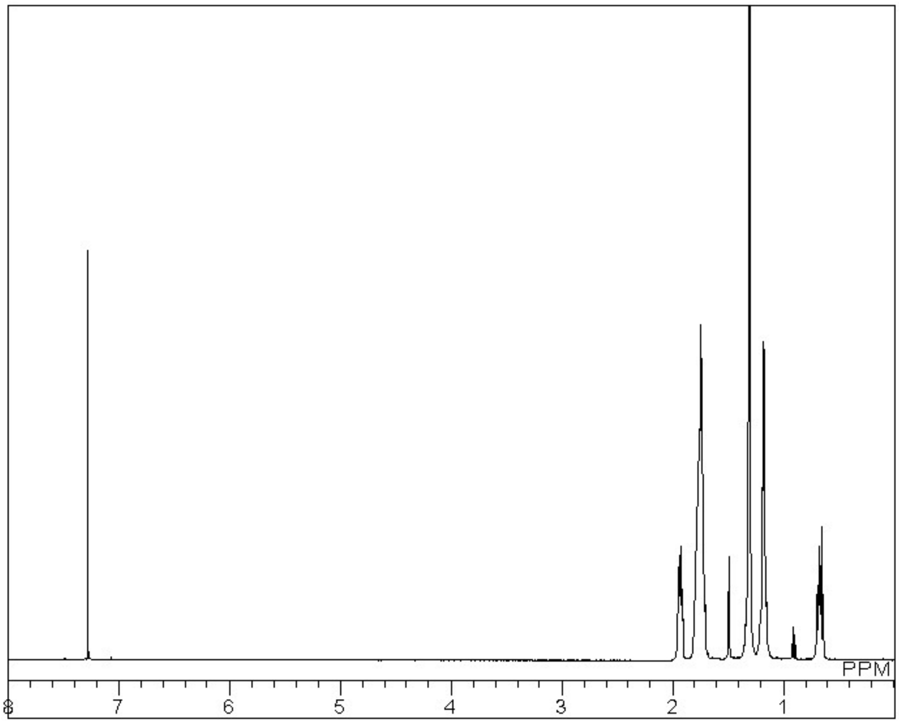

Supplementary Figure 27. ^{1}H NMR spectrum of the H-poly(NB) from **5** shown in **Supplementary Table 10** (recorded in $CDCl_3$ at 27 °C).

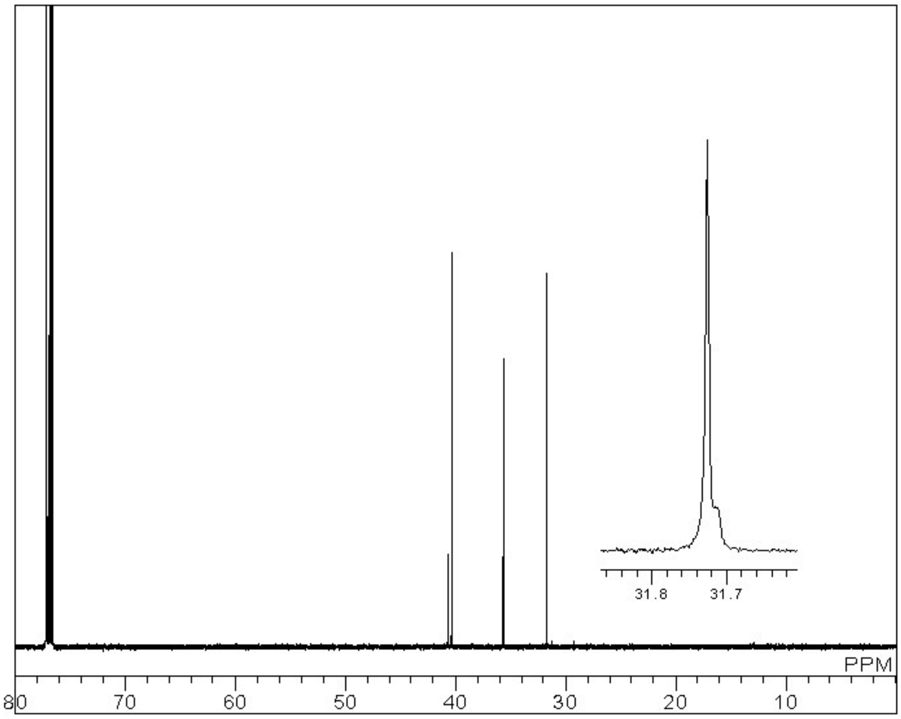

Supplementary Figure 28. ^{13}C NMR spectrum of the H-poly(NB) from **5** shown in **Supplementary Table 10** (recorded in $CDCl_3$ at 60 °C).

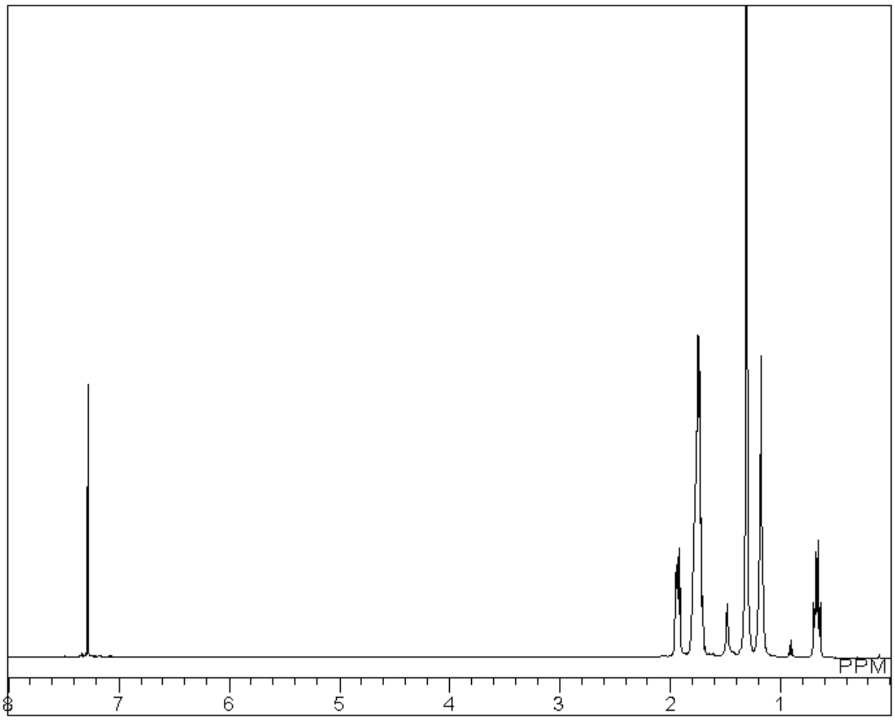

Supplementary Figure 29. ^{1}H NMR spectrum of the syndio-H-poly(NB) from **9** shown in **Supplementary Table 10** (recorded in $CDCl_3$ at 27 °C).

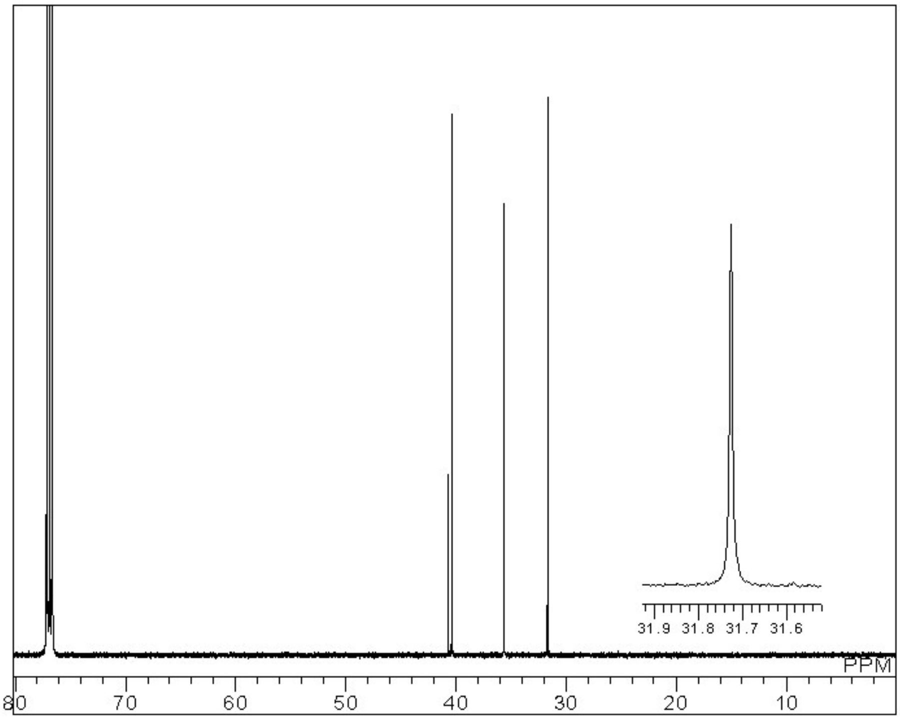

Supplementary Figure 30. ^{13}C NMR spectrum of the H-poly(NB) from **9** shown in **Supplementary Table 10** (recorded in $CDCl_3$ at 60 °C).

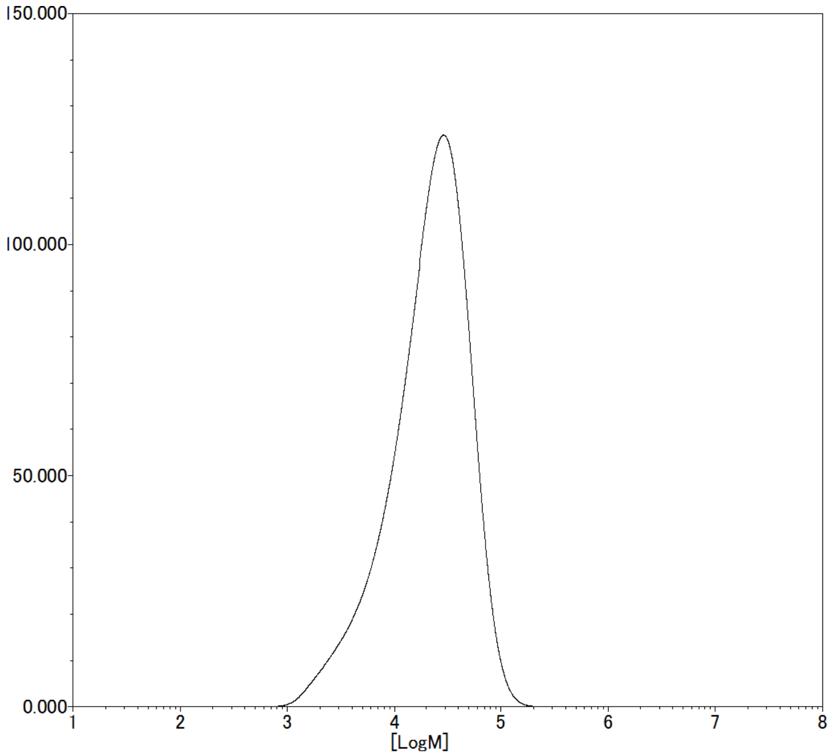

Supplementary Figure 31. GPC trace of the poly(NB) from **1** shown in **Supplementary Table 10**.

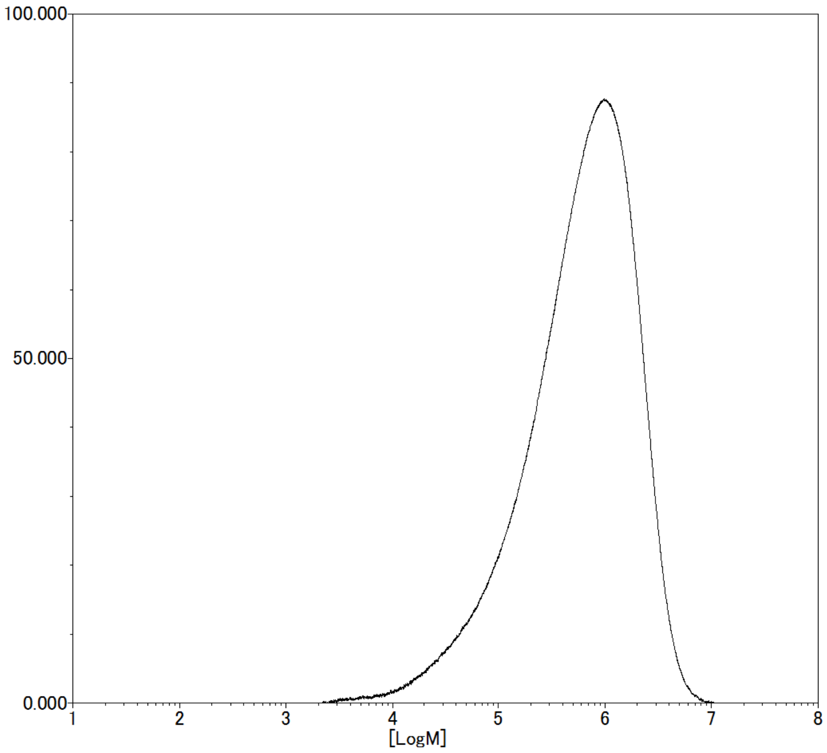

Supplementary Figure 32. GPC trace of the *cis*-, syndio-poly(NB) from **9** shown in **Supplementary Table 10**.

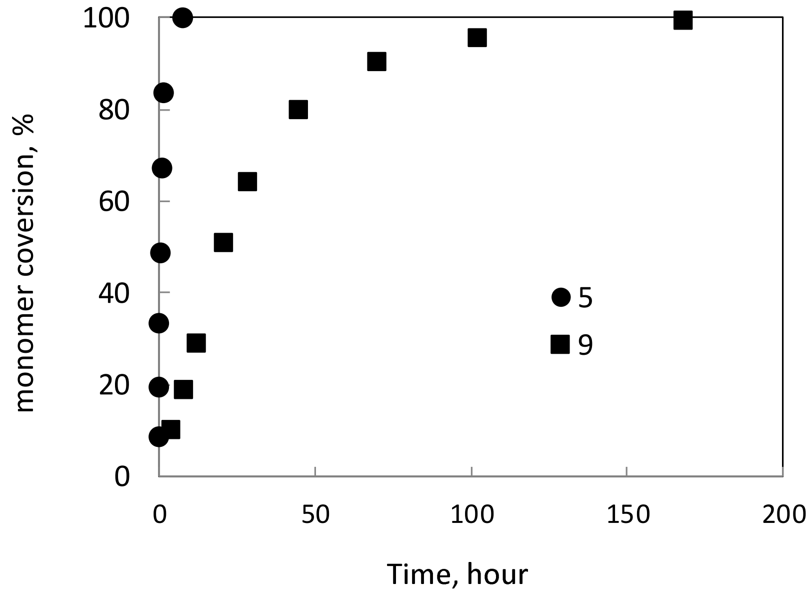

Supplementary Figure 33. Time profiles of the polymerization of NB by **5** and **9** shown in **Supplementary Table 10**.

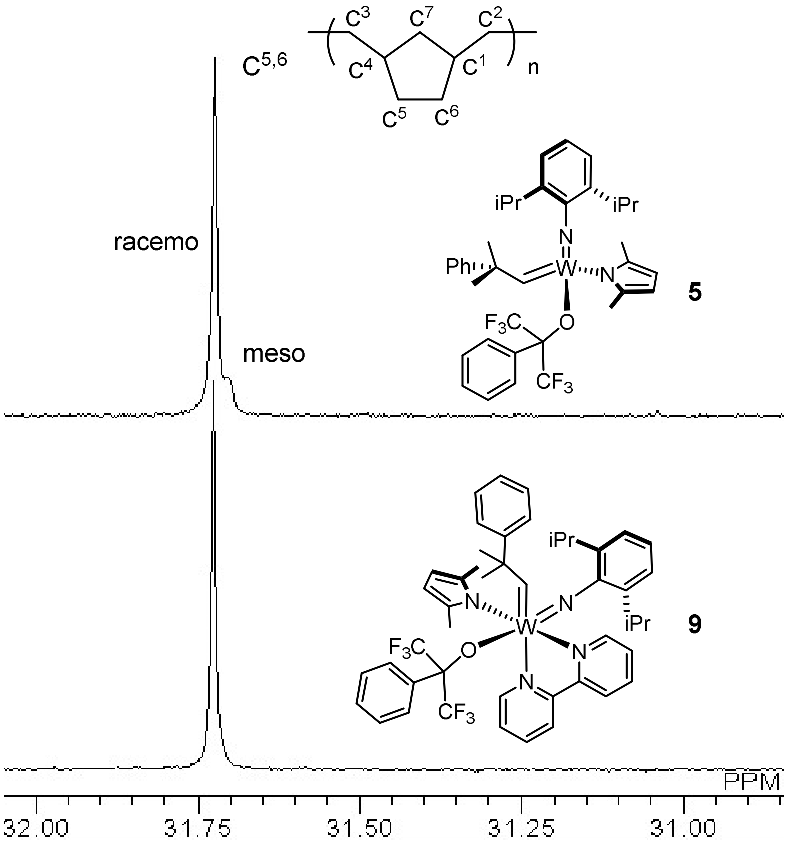

Supplementary Figure 34. Selected ^{13}C NMR spectra of the H-poly(NB)s from **5** and **9** shown in **Supplementary Table 10**, **Supplementary Figure 28** and **Supplementary Figure 30** (recorded in $CDCl_3$ at 60 °C).

6. Supplementary References

[1] Heppekausen, J. & Fürstner A. Rendering Schrock-type Molybdenum Alkylidene Complexes Air Stable: User-Friendly Precatalysts for Alkene Metathesis. *Angew. Chem. Int. Ed.* **50**, 7829–7832 (2011).

[2] Bailey B. C., Schrock, R. R., Kundu, S., Goldman, A. S., Huang, Z. & Brookhart M. Evaluation of Molybdenum and Tungsten Metathesis Catalysts for Homogeneous Tandem Alkane Metathesis. *Organometallics* **28***,* 355–360 (2009).

[3] Chai, J.-D. & Head-Gordon, M. Long-range corrected hybrid density functionals with damped atom–atom dispersion corrections. *Phys. Chem. Chem. Phys.* **10**, 6615–6620 (2008)

[4] Chai, J.-D. & Head-Gordon, M. Systematic optimization of long-range corrected hybrid density functionals. *J. Chem. Phys.* **128**, 084106 (2008).

[5] Grimme, S. Semiempirical GGA‐type density functional constructed with a long‐range dispersion correction. *J. Comput. Chem.* **27**, 1787–1799 (2006).

[6] For the Def2SVP and Def2TZVPP basis sets, see: Weigend, F. & Ahlrichs, R. Balanced basis sets of split valence, triple zeta valence and quadruple zeta valence quality for H to Rn: Design and assessment of accuracy. *Phys. Chem. Chem. Phys.* **7**, 3297–3305 (2005).

[7] Koh, M. J. *et al.* Molybdenum chloride catalysts for *Z*-selective olefin metathesis reactions. *Nature* **542**, 80-85 (2017).

[8] Marenich, A. V., Cramer, C. J. & Truhlar, D. G. Universal Solvation Model Based on Solute Electron Density and on a Continuum Model of the Solvent Defined by the Bulk Dielectric Constant and Atomic Surface Tensions. *J. Phys. Chem. B,* **113**, 6378–6396 (2009).

[9] Boys, S. F. & Bernardi, F. The calculation of small molecular interactions by the differences of separate total energies. Some procedures with reduced errors. *Mol. Phys* **19**, 553–566 (1970).

[10] *Gaussian 16*, *Revision A.03*, M. J. Frisch, G. W. Trucks, H. B. Schlegel, G. E. Scuseria, M. A. Robb, J. R. Cheeseman, G. Scalmani, V. Barone, B. Mennucci, G. A. Petersson, H. Nakatsuji, M. Caricato, X. Li, H. P. Hratchian, A. F. Izmaylov, J. Bloino, G. Zheng, J. L. Sonnenberg, M. Hada, M. Ehara, K. Toyota, R. Fukuda, J. Hasegawa, M. Ishida, T. Nakajima, Y. Honda, O. Kitao, H.

Nakai, T. Vreven, J. A. Montgomery, J. E. Peralta, F. Ogliaro, M. Bearpark, J. J. Heyd, E. Brothers, K. N. Kudin, V. N. Staroverov, R. Kobayashi, J. Normand, K. Raghavachari, A. Rendell, J. C. Burant, S. S. Iyengar, J. Tomasi, M. Cossi, N. Rega, J. M. Millam, M. Klene, J. E. Knox, J. B. Cross, V. Bakken, C. Adamo, J. Jaramillo, R. Gomperts, R. E. Stratmann, O. Yazyev, A. J. Austin, R. Cammi, C. Pomelli, J. W. Ochterski, R. L. Martin, K. Morokuma, V. G. Zakrzewski, G. A. Voth, P. Salvador, J. J. Dannenberg, S. Dapprich, A. D. Daniels, O. Farkas, J. B. Foresman, J. V. Ortiz, J. Cioslowski, D. J. Fox, Gaussian, Inc., Wallingford CT, 2016.

[11] *MacroModel*, Schrödinger, LLC, New York, NY, 2016.
